# Proteome‐based molecular subtyping and therapeutic target prediction in gastric cancer

**DOI:** 10.1002/1878-0261.13654

**Published:** 2024-04-16

**Authors:** Changyuan Hu, Jiangning Song, Terry Kwok, Elizabeth V. Nguyen, Xian Shen, Roger J. Daly

**Affiliations:** ^1^ Cancer Program, Biomedicine Discovery Institute Monash University Clayton Australia; ^2^ Department of Biochemistry and Molecular Biology Monash University Clayton Australia; ^3^ Wenzhou Medical University‐Monash BDI Alliance in Clinical and Experimental Biomedicine Wenzhou Medical University China; ^4^ Infection and Immunity Program, Monash Biomedicine Discovery Institute Monash University Clayton Australia; ^5^ Department of Microbiology Monash University Clayton Australia; ^6^ Department of Gastrointestinal Surgery, The First Affiliated Hospital Wenzhou Medical University China

**Keywords:** gastric cancer, kinomics, mass spectrometry, precision oncology, proteomics

## Abstract

Different molecular classifications for gastric cancer (GC) have been proposed based on multi‐omics platforms with the long‐term goal of improved precision treatment. However, the GC (phospho)proteome remains incompletely characterized, particularly at the level of tyrosine phosphorylation. In addition, previous multiomics‐based stratification of patient cohorts has lacked identification of corresponding cell line models and comprehensive validation of broad or subgroup‐selective therapeutic targets. To address these knowledge gaps, we applied a reverse approach, undertaking the most comprehensive (phospho)proteomic analysis of GC cell lines to date and cross‐validating this using publicly available data. Mass spectrometry (MS)‐based (phospho)proteomic and tyrosine phosphorylation datasets were subjected to individual or integrated clustering to identify subgroups that were subsequently characterized in terms of enriched molecular processes and pathways. Significant congruence was detected between cell line proteomic and specific patient‐derived transcriptomic subclassifications. Many protein kinases exhibiting ‘outlier’ expression or phosphorylation in the cell line dataset exhibited genomic aberrations in patient samples and association with poor prognosis, with casein kinase I isoform delta/epsilon (CSNK1D/E) being experimentally validated as potential therapeutic targets. Src family kinases were predicted to be commonly hyperactivated in GC cell lines, consistent with broad sensitivity to the next‐generation Src inhibitor eCF506. In addition, phosphoproteomic and integrative clustering segregated the cell lines into two subtypes, with epithelial–mesenchyme transition (EMT) and proliferation‐associated processes enriched in one, designated the EMT subtype, and metabolic pathways, cell–cell junctions, and the immune response dominating the features of the other, designated the metabolism subtype. Application of kinase activity prediction algorithms and interrogation of gene dependency and drug sensitivity databases predicted that the mechanistic target of rapamycin kinase (mTOR) and dual specificity mitogen‐activated protein kinase kinase 2 (MAP2K2) represented potential therapeutic targets for the EMT and metabolism subtypes, respectively, and this was confirmed using selective inhibitors. Overall, our study provides novel, in‐depth insights into GC proteomics, kinomics, and molecular taxonomy and reveals potential therapeutic targets that could provide the basis for precision treatments.

## Graphical abstract

In this study we exploit a large panel of gastric cancer (GC) cell lines and powerful (phospho)proteomic workflows to provide the most comprehensive characterization of the GC proteome to date. The data are used to generate novel subclassifications and we identify, and then validate through functional analyses and interrogation of publicly available data, kinase targets for broad or precision GC treatment.

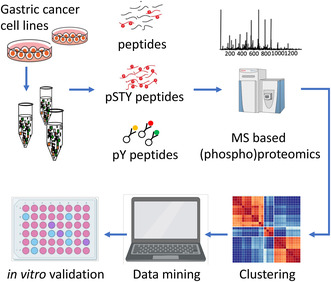

**Abbreviations**
ACN: acetonitrile
ACRG: Asian Cancer Research Group
CCLE: the Cancer Cell Line Encyclopedia
CIN: chromosomal instability
DDA: data‐dependent acquisition
DIA: data‐independent acquisition
DMSO: dimethyl sulfoxide
EBV: epstein–barr virus
EMT: epithelial‐mesenchyme transition
FBS: fetal bovine serum
FDR: false discovery rate
GC: gastric cancer
G‐Dif: genomic diffuse
G‐Int: genomic intestinal
GO: gene ontology
GS: genomically stable
GSEA: gene set enrichment analysis
HRM: hyper reaction monitoring
IAP: immunoaffinity purification
INKA: Integrative Inferred Kinase Activity
KEGG: Kyoto Encyclopedia of Genes and Genomes
KSEA: kinase set enrichment analysis
KUGH: Kosin University Gospel Hospital
MAD: mean absolute deviation
MLR: multilinear regression
MS: mass spectrometry
MSI: microsatellite instability
MTS: 3‐(4,5‐dimethylthiazol‐2‐yl)‐5‐(3‐carboxymethoxyphenyl)‐2‐(4‐sulfophenyl)‐2H‐tetrazolium
NMF: non‐negative matrix factorization
PBS: phosphate‐buffered saline
PP: phosphoproteome
PTM‐SEA: post‐translational modifications signature enrichment analysis
PUCH: Peking University Cancer Hospital
RPMI: Roswell Park Memorial Institute
SFK: Src family kinase
TBS: tris‐buffered saline
TCEP: tris (2‐carboxyethyl) phosphine hydrochloride
TCGA: The Cancer Genome Atlas
TFA: trifluoroacetic acid
TME: tumor microenvironment
WGCNA: weighted correlation network analysis
WP: whole proteome
YP: tyrosine phosphorylation

## 1 Introduction

Gastric cancer (GC) ranks fifth for morbidity and fourth for mortality across all cancers globally [1], presenting a dismal prognosis with 5‐year overall survival rates between 5.6% and 81% for different cancer stages [2, 3]. As most patients with GC are still treated in a ‘one‐size‐fits‐all’ manner, approaches capable of classifying a heterogeneous population of GC into homogenous subtypes to better inform treatment strategies and/or predict prognosis are urgently required. However, classifications based on the tumors' anatomical location [4], clinical staging [3], or histopathological features [5] are not able to inform precision treatment [6, 7].

Reflecting this need, molecular subgroupings have emerged in the last decade, with the goal of identifying prognostic and predictive biomarkers that inform the selection of patient populations for optimal treatment. The Cancer Genome Atlas (TCGA) project characterized GC into four clusters based on integrative clustering using genomic data: Epstein–Barr virus (EBV)‐positive, microsatellite instability (MSI), genomically stable (GS) and chromosomal instability (CIN), initiating the age of omics subclassification of GC [8]. Recently, four subgroups of GC, each associated with specific signaling pathways were identified based on mRNA, protein, phosphoproteome, and glycoproteome profiles. These subsets were characterized by cell proliferation‐related processes, immune response, metabolism pathways, and invasion, respectively [9]. Several other studies also focused on the molecular subclassification of GC based on primary tumors [10, 11, 12, 13, 14]. Although these sophisticated and comprehensive studies have provided an extensive understanding of GC molecular subtypes, they have yet to directly benefit patient prognosis or guide treatment strategies.

Protein kinases have emerged as major targets for the precision treatment of many malignancies, and it is likely that new kinase‐based therapeutic modalities will emerge by identifying new oncogenic roles for existing therapeutic targets, as well as novel kinase cancer drivers [15]. As proof‐of‐principle, Trastuzumab, targeting the proto‐oncogene ERBB2, is the first targeted approach approved by the FDA for first‐line treatment of advanced GC [16]. Furthermore, results from the VIKTORY Umbrella trial support patient subclassification into biomarker groups based on genomic sequencing and assignment of patients to corresponding kinase‐targeted therapies. In general, this biomarker‐driven approach resulted in a better prognosis than conventional chemotherapy [17]. However, only ~ 20% of patients with GC currently exhibit an increase in survival time of 3 months or more following targeted therapy [17, 18, 19], highlighting the need for improved precision treatment strategies.

Reflecting the fundamental role of protein kinases in the regulation of protein phosphorylation, the extension of genomic analyses to mass spectrometry (MS)‐based characterization of the global phosphoproteome represents a powerful strategy for detecting aberrant activation of specific kinases and their downstream pathways in cancer, complementing genomics in identifying therapeutic strategies and molecular taxonomies [15, 20]. Indeed, several (phospho)proteomic datasets for GC have been generated and utilized in this manner [9, 13, 14]. However, most of the phosphosites identified by standard phosphoproteomic analyses represent a modification of serine or threonine residues rather than tyrosine, which is a concern given the high representation of tyrosine kinases among known kinase cancer drivers [15]. Given the relatively low abundance of tyrosine phosphorylation in the proteome, and hence the larger sample size required for comprehensive tyrosine phosphorylation profiling, this indicates that cell line‐based profiling, where scale‐up is straightforward, may bear fruit and reveal novel targets in GC. In addition, this approach enables rapid testing of candidates in functional assays to identify high‐priority targets that can be validated by interrogation of publicly available clinical data.

In this study, we exploit a large panel of GC cell lines and three powerful (phospho)proteomic workflows, including tyrosine phosphorylation profiling, to provide the most comprehensive characterization of the GC proteome to date. The resulting datasets are used individually and in an integrated manner to generate novel subclassifications with subtype‐specific molecular features. In addition, we identify, and then validate through functional analyses and interrogation of publicly‐available data, potential kinase targets for broad or precision treatment of GC. Overall, our work provides novel and important insights into GC biology and highlights several kinases as the foundation for precision treatment.

## 2 Materials and methods

### 2.1 Cell lines

The TMK1 (RRID: CVCL_4384), MKN28 (RRID: CVCL_1416), SNU601 (RRID: CVCL_0101), N87 (NCI‐N87 (RRID: CVCL_1603)), AGS (RRID: CVCL_0139), and AZ521 (RRID: CVCL_2862) GC cell lines were obtained from Professor Brendan Jenkins (Hudson Institute of Medical Research, Clayton, Australia). Of note, AZ521 is a duodenal adenocarcinoma cell line, but since pyloric gland‐type adenocarcinoma of the duodenum showing gastric‐type differentiation has been reported [21], we included this cell line for proteome analysis, consistent with the DepMap and the Cancer Cell Line Encyclopedia (CCLE) databases classifying it as GC under the disease category. SNU1750 (RRID: CVCL_8914), YCC3 (RRID: CVCL_9657), KatoIII (RRID: CVCL_0371), and Ocum1 (RRID: CVCL_3084) GC cell lines were obtained from Associate Professor Sefi Rosenbuluh (Monash University, Clayton, Australia). The AGS‐EBv (eAGS) [22] and YCCEL1 (RRID: CVCL_L440) GC cell lines were a gift from Professor Lawrence Young (Warwick University, Coventry, UK). NCC24 (SNU‐NCC‐24 (RRID: CVCL_8899)) and SNU719 (RRID: CVCL_5086) GC cell lines were purchased from the Korean Cell Line Bank. Their clinical characteristics and molecular features are listed in Table S1. All GC cell lines were cultured in Roswell Park Memorial Institute (RPMI) 1640 media supplemented with 10% (v/v) fetal bovine serum (FBS). RPMI 1640 media was obtained from Monash Biomedicine Discovery Institute's media center, and FBS was purchased from Gibco (Grand Island, NY, USA). Authentication of all cell lines was conducted within the last 3 years using a combination of techniques by CellBank Australia, including short tandem repeat polymorphism, single‐nucleotide polymorphism, and fingerprint analyses. This approach involves utilizing tetranucleotide or pentanucleotide repeats within established loci to create a unique profile for each cell line, which is then cross‐referenced against other stocks of the same cell line. Typically, cell lines are deemed matching if their profiles show more than 80% similarity. The cells also underwent routine mycoplasma testing by PCR. Cells were maintained at 37 °C in a 5% CO_2_ atmosphere. The culture medium was replaced with fresh medium every 2–3 days. To passage cells, cells were washed twice with 1× phosphate‐buffered saline (PBS), detached from dishes with 0.05% (w/v) trypsin/ethylenediaminetetraacetic acid (EDTA) (Gibco) in the incubator for 5 ~ 10 min, and passaged at a 1 : 4 ratio.

### 2.2 Cell lysate preparation for MS proteomics

Sub‐confluent (80–90%) cells were removed from the incubator and immediately placed on ice, followed by two washes with chilled 1× Tris‐buffered saline (TBS) (Merck, Burlington, MA, USA). For each 15 cm dish, cells were lysed with 500 μL sodium deoxycholate (Merck) lysis buffer [23], the sample was heated at 95 °C for 5 min and sonicated at 10 amps for 1 min, which was repeated 3×. Cell lysates were spun down at 33 000 g at 4 °C for 20 min, and the supernatant was transferred to new 15 mL Falcon® tubes. Supernatant (10 μL) of each cell line was transferred into new 1.5 mL Eppendorf® tubes for protein quantitation by the bicinchoninic acid assay (Pierce™ BCA protein assay kit, Thermo Fisher Scientific, Waltham, MA, USA) according to the user instructions.

### 2.3 Protein digestion

The lysates were normalized for protein concentration, denatured using Tris (2‐carboxyethyl) phosphine hydrochloride (TCEP) (Merck) (5 mm) by shaking at 220 r.p.m. for 15 min at room temperature, followed by alkylation. To achieve alkylation, lysates were incubated with 10 mm iodoacetamide for 30 min in the dark at room temperature with shaking at 220 r.p.m. Lys‐C (Wako Chemicals, Osaka, Japan) and trypsin (Promega, Madison, WI, USA), at an enzyme‐protein ratio of 1 : 100 (w/w), were added into the lysates for overnight digestion at 37 °C with shaking at 220 r.p.m. The digested lysates were then acidified with trifluoroacetic acid (TFA) (Merck) (2% v/v) to remove sodium deoxycholate. After vortexing for 1 min and centrifuging at 15 000 ***g*** for 10 min, the supernatant was kept, and this acid‐mediated cleaning step was repeated again. On completion, the peptide solution was divided into two parts for the determination of whole proteome (WP) and tyrosine phosphorylation (YP) profiling.

The lysate preparation for phosphoproteome (PP) followed the EasyPhos protocol [23]. Briefly, the normalized lysates were denatured and alkylated simultaneously using a buffer containing 100 mm TCEP and 400 mm 2‐chloroacetamide, followed by the same enzymatic digestion as above. However, an isopropanol mixture step instead of TFA‐mediated cleaning step was performed to prevent the acid‐induced precipitation of sodium deoxycholate.

### 2.4 C18 StageTips‐based desalting for whole proteomics

A certain volume of each peptide mixture (equal to 100 μg protein starting material) was acidified with 10% formic acid to pH 2 ~ 3. C18 StageTips were activated using 200 μL methanol and equilibrated using 200 μL 80% acetonitrile (ACN) and 0.1% TFA (v/v) buffer. After loading the peptide mixtures onto the top of StageTips, the columns were centrifuged at 500 ***g*** until the entire solution flowed through the C18 membrane, which was followed by two washes with 200 μL 0.1% TFA. After sample elution, the desalted peptides were then dried in a SpeedVac concentrator and resuspended in MS loading buffer (2% ACN, 0.3% TFA (v/v)) containing hyper reaction monitoring (HRM™) peptide mix (Biognosys, Schlieren, Switzerland) for MS analysis.

### 2.5 Phosphopeptide enrichment and desalting

A certain volume of each peptide mixture with isopropanol described above (equal to 500 μg protein starting material) was mixed with EasyPhos enrichment buffer and centrifuged to collect the supernatants [23]. Samples were mixed with titanium dioxide beads (GL Sciences, Tokyo, Japan) at a 12 : 1 (w/w) bead to protein ratio, and incubated for 5 min at 40 °C with shaking at 2000 r.p.m. followed by four washes. Phosphopeptides were eluted from the beads and dried in the SpeedVac. After resuspended onto the Empore™ SDB–RPS StageTips (Sigma‐Aldrich, Kawasaki, Kanagawa, Japan), the phosphopeptides were then eluted with StageTip elution buffer, dried completely using the SpeedVac, and resuspended in 8 μL MS loading buffer with HRM™ peptide mix ready for MS analysis.

### 2.6 Phosphorylated Tyr peptide preparation

A certain volume of each peptide mixture (equal to 20 mg of protein starting material) was loaded onto ACN‐activated Sep‐Pak® columns, washed with 12 mL of 0.1% TFA, and eluted with 50% ACN and 0.2% TFA buffer (v/v). The frozen eluents were lyophilized for 1 ~ 2 days. Meanwhile, Rec‐Protein G Invitrogen® (Waltham, MA, USA) beads and antibodies (1 : 4 (v/w) ratio) were prepared. Briefly, the Invitrogen® beads were incubated with 50 μL phosphor‐Tyr‐1000 antibody (Cell Signaling, Danvers, MA, USA) and 50 μL phosphor‐Tyr‐20 antibody (BD Bioscience, Franklin Lakes, NJ, USA) in 1.7 mL of immunoprecipitation binding buffer (50 mm MOPS, 10 mm Na_2_HPO_4_, 50 mm NaCl (pH 7.5) overnight at 4 °C). The next day, the coupled beads were washed with 1.8 mL of immunoaffinity purification (IAP) washing buffer (50 mm Tris–HCl, 150 mm NaCl, 1% n‐octyl‐b‐d‐glucopyranoside (pH 7.4)) for three times and transferred into new 2 mL Eppendorf® tubes. The lyophilized peptides dissolved in 1.8 mL of IAP washing buffer (close to neutral pH) were combined with the bead slurry and incubated overnight at 4 °C. The beads were washed three times with 1 mL of IAP washing buffer, which was followed by three washes of 1 mL of milli Q water. Following the last wash, the phosphorylated tyrosine peptides were eluted using 55 μL of 0.15% trifluoroacetic acid, followed by shaking at 1400 r.p.m. for 15 min at room temperature, after which the supernatant was collected. This process was repeated twice for the phosphorylated tyrosine peptides to be eluted in a final volume of 110 μL trifluoroacetic acid. The phosphorylated tyrosine peptides mixture was next desalted following the steps described in C18 StageTips‐based desalting for whole proteomics and resuspended in MS loading buffer with HRM peptide mix in preparation for MS analysis.

### 2.7 Mass spectrometry‐based (phospho)proteome analysis

The triplicated WP and PP samples were analyzed on an UltiMate 3000 RSLC nano‐LC system (Thermo Fisher Scientific) coupled to a Q Exactive HF Mass Spectrometer (Thermo Fisher Scientific). The peptides were loaded via a Pepmap100 trap column, 100 μm × 2 cm nanoviper, (Thermo Fisher Scientific), eluted and separated on a Pepmap100 RSLC analytical column, 75 μm × 50 cm, nanoViper, C18 (Thermo Fisher Scientific). For each injection, 1 μg of peptides was loaded onto the column and eluted using increasing concentrations of buffer B (80% ACN/0.1% formic acid) at 250 nL·min^−1^ over 150 min. The eluent was nebulized and ionized using a nanoelectrospray source (ThermoFisher Scientific) with a distal‐coated fused silica emitter (Trajan, Ringwood, Victoria, Australia). The capillary voltage was set at 1.7 kV. For WP and PP, the HRM‐data‐independent acquisition (DIA) mode consisted of a MS1 scan (scan range from 370 to 2000 m/z, at resolution of 30 000 (at m/z 200), an AGC target of 1 × 10^6^ and a maximum ion injection time of 50 ms) followed by MS2 scans with 60 DIA windows at a resolution of 15 000 (at m/z 200), an AGC target of 2 × 10^5^ with automatic injection time, isolation window of 10 m/z (Table S2A). The stepped collision energies were 22.5%, 25%, and 27.5%. The spectra were recorded in profile type.

In terms of replicated samples for immunoprecipitated YP (profiled by Q Exactive Plus (Thermo Fisher Scientific)), in addition to spectral libraries for WP and PP (profiled by Q Exactive HF), the mass spectrometer was operated in the data‐dependent acquisition (DDA) mode to automatically switch between full MS scans and tandem MS/MS scans. Survey full scan MS spectra (m/z 300–1750) were acquired in the Orbitrap with 70 000 resolution (at m/z 200) after accumulation of ions to a 1 × 10^6^ target value with a maximum injection time of 30 ms. Up to 10 of the most intensely charged ions (z ≥ +2) were sequentially isolated and fragmented in the collision cell by higher‐energy collisional dissociation with the following parameters, fixed injection time of 120 ms, 17 500 resolution and automatic gain control target of 1 × 10^5^. Dynamic exclusion was set to 30 s for PP and YP and 15 s for WP.

### 2.8 DDA/HRM™‐DIA data searching

All raw phosphorylated tyrosine profiling files from DDA acquisition were analyzed with maxquant (version 1.6.0.16) to obtain phosphorylated tyrosine peptides [24]. Database searching was performed with the following settings: carbamidomethylation as a fixed modification; oxidation, N‐terminal acetylation, and phosphorylation (Ser, Thr, Tyr) as variable modifications; up to two missed cleavages permitted; and 1% false discovery rate (FDR) for protein and peptide identification. The human protein sequence databases were downloaded from https://www.uniprot.org/.

In terms of DIA data analysis, spectral libraries for WP or PP were generated with similar settings for the DDA data searching approach in spectronaut® software (version 13.12.200217.43655, Biognosys), separately. The multiple DIA datafiles for each sample were transformed into htrms format using the HTRMS Converter (Biognosys) and then imported into spectronaut® software for generating WP or PP dataset. All the parameters for phosphopeptide and/or protein identification and quantification were retained as default. Before exporting the data to the Excel spreadsheet, quantified proteins or phosphopeptides with a *q*‐value greater than 0.01 were excluded from further analysis. Furthermore, the matrices of phosphopeptides were converted to phosphosite‐centric matrices by summing the phosphorylation abundance of the same phosphorylation site on multiple phosphorylated peptides.

### 2.9 Data processing

Quantile normalization was applied to three datasets. The missing values in datasets of WP, PP, and YP were imputed using the spectronaut® software, r package phosr [25], and Windows® software perseus [26], respectively. The batch effect in each dataset was evaluated and removed via the R package probatch [27] if the batch effect was present. The reproducibility of each dataset was evaluated by Pearson's correlation analysis.

### 2.10 Subtype identification

To identify subtypes using an individual dataset, the molecules (proteins or phosphosites) showing significant variations in their abundance across the cell line panel were first selected by calculating the mean absolute deviation (MAD) for each protein across the whole cell line. We filtered the proteins with the top 10%, 20%, 30%, and 50% of MADs as input datasets for evaluating the efficiency of classification and evaluated the robustness of the subgrouping results using a cophenetic correlation coefficient by performing non‐negative matrix factorization (NMF) [28] clustering 100 times. The final parameters of each dataset were determined as the best combination of cluster number *k* and MAD that resulted in the most stable *k*‐cluster decomposition. As a result, 568 proteins with the top 10% of MADs and *k* = 3 were settled for WP dataset; 1691 phosphosites with top 20% of MADs and *k* = 2 were determined for PP dataset; 4780 phosphorylated tyrosine sites with all MADs and *k* = 3 were settled for YP dataset. The consistency of subclassifications was further confirmed by checking the cluster‐specific patterns through the NMF consensus matrix, as well as the similarity within and across the subclusters through silhouette values. Characteristic proteins of each WP cluster were also obtained by the NMF clustering.

For integrative clustering analysis, the subtypes of each sample identified from subclassification of the WP, PP, and YP datasets were first converted to ones and zeros with ones representing that the corresponding samples belonged to the subtype. Thus, this subgrouping information could be transformed into a *n* × *m* (subtypes by samples) 1‐0 binary table. Subsequently, the R package consensusclusterplus [29] was introduced to perform integrative clustering 500 times on the 1‐0 binary table.

### 2.11 Functional annotation

To address the subcellular distribution of the quantified molecules, cellular components from gene ontology (GO) was applied to the phospho/proteomic data sets. The protein module were determined by the R package wgcna [30]. The differential analysis across subsets was performed using the R package limma [31] or repeated measure analysis of variance (ANOVA). To control the FDR of multiple comparisons, *P* values were adjusted by the Benjamin–Hochberg test. To draw a landscape of molecular function for the GC cell line panel, three different functional annotation approaches were adopted: (a) gsea against additional defined gene sets from the Broad Institute was conducted via software gsea (version 4.0.3) [32]; (b) Gene ontology analysis was directly applied against differentially expressed or phosphorylated proteins on http://geneontology.org/ [33]; (c) The PP data were also explored by post‐translational modifications signature enrichment analysis (PTM–SEA) on the webserver from Broad Institute GenePattern [34].

### 2.12 Kinase activity prediction

The phosphosite‐centric YP matrix was uploaded into the online InKA server for making the activity prediction. InKA can return a sample‐centric kinase activation list, which contains whole kinases detected in the corresponding sample that are ordered by InKA activity scores [35].

For the phosphosite‐centric PP dataset, the raw and normalized (i.e., substrate p‐site intensity/protein intensity) PP matrix was calculated for predicting kinase activities. Three kinase activation predicting approaches including mean fold difference, kinase set enrichment analysis (KSEA), and the multilinear regression (MLR) model in the R package phosmap [36] were adopted for all detected substrate sites of the same kinase as a measurement of the kinase activation/inhibition. Thus, six PP kinase activity results were generated by the different combination of normalized and raw phosphosite‐centric PP dataset and the use of three prediction approaches. The difference in kinase activities between the two subsets was compared using these six kinase activity matrices and recorded. The threshold was defined as the *P* value < 0.05 while absolute log2FC > 1. The number of times a kinase was assigned to either of the two subgroups using the six activity matrices was then assessed as a measure of differential activity for that kinase between the two subsets.

### 2.13 Public database data mining

A 40‐kinase expression signature was used to subcategorize GC cell lines in the DepMap [37] and genomics of drug sensitivity in cancer (GDSC) database [38]. Drug responses (IC50 value of compounds) and viability changes after gene knockdown (CRISPR gene dependence score of kinase) of subset‐specific GC cell lines were compared using these datasets. To identify subset‐specific compounds, the following criteria were utilized: a compound with a *P* value < 0.05, and absolute log2 Fold Change (log2FC) > 1, was assigned as subset‐specific; otherwise, the compound was regarded as equal. To determine subset‐specific lethal kinases, the criterion was slightly modified. A kinase with a *P* value < 0.1 and absolute log2FC > 2, was defined as subset‐specific; otherwise, the kinase was regarded as having no difference.

Three independent GC cohorts (TCGA, ACRG, and PUCH) were included and reassigned into EMT and metabolism subgroups according to a machine learning model. The mutation variations of patients with GC were obtained and plotted using https://www.cbioportal.org/ [39].

### 2.14 Machine learning model prediction

Using the built‐in NMF algorithm, the most representative set of proteins was first extracted from the WP data to serve as the metagene for each WP subgroup. A total of 42 proteins, serving as the simplified WP features, were included in the next steps of model training and prediction. Using the scikit‐learn software package implemented in python 3.0 (Python Software Foundation, Wilmington, DE, USA), the 42‐feature protein expression profiles of the labeled (EMT, cell proliferation/EMT or metabolism) cell line panel was firstly split into a training and validation cohort using a 9 : 1 ratio, and then trained using a Neural Networks learning algorithm. The model can assign patients GC from clinical cohorts into different subgroups against their 42‐gene profiling data. To obtain a robust prediction result, each model predicted 500 times for each patient with GC. Only patients' results with > 33% consistency meaning that the model assigned greater than 166 times in a specific subgroup were kept for further analysis.

### 2.15 siRNA knockdown

GC cells at 3 × 10^3^ cells per well in 96‐well plates were reverse transfected with 0.15 μL of DharmaFECT1 (Dharmacon RNAi Technologies, Horizon Discovery, Waterbeach, United Kingdom) and siGENOME siRNAs (Human ERBB2 siRNA, #MQ‐003126‐04‐0020, Human FGFR2 siRNA, #MQ‐003132‐04‐0020) at a final concentration of 20 nm. Media were changed at 24 and 96 h and the experiment ended at 144 h post transfection. Cell proliferation assays was performed at 24, 48, 96, and 144 h.

### 2.16 Inhibitor treatment and activation of autophagy

Inhibitors listed in Table S2B, which include CSNK1D/E inhibitor SR3029, ErbB family inhibitor Lapatinib, pan‐PI3K inhibitor BKM120, AKT inhibitor MK2206, Src kinase family inhibitor eCF506, mTOR inhibitor Rapamycin, and MEK inhibitor Trametinib were purchased from Selleckchem and reconstituted in dimethyl sulfoxide (DMSO) (Merck).

In each well of the 96‐well plates, a total of 3 × 10^3^ cells were seeded in 80 μL of complete media. After 24 h, the cells were treated with 20 μL of complete media containing either inhibitors at their indicated concentrations or DMSO (negative control). The experiment was continued 96 h post drug treatment and then cell proliferation was measured. For colony formation assays, 300 cells per well, in 300 μL complete media were seeded into 24‐well plates. After 24 h, the media was replaced with complete media containing either DMSO (negative control) or Src kinase family inhibitor eCF506 at the indicated concentration. Media ± drug was replaced every 96 h until colonies were evident.

For starvation‐induced autophagy experiments, cells were either maintained in growth media or washed twice with PBS and switched to nutrient‐free Earle's Balanced Salts media (Sigma‐Aldrich) for 4 h. For autophagy inhibition, cells were starved as above with the addition of 100 nm bafilomycin A1 (Sigma‐Aldrich) or DMSO vehicle [40].

### 2.17 Effect of SR3029 on CSNK1D/E phosphorylation

MKN28 cells were treated with 10 nm SR3029 for 48 h. Post‐treatment, cell lysates underwent EasyPhos protocol processing. Two biological replicates were analyzed by MS using DIA and DDA, respectively. Phosphorylation at CSNK1D S384 was identified in the result matrix, which was searched using spectronaut® or maxquant in accordance with the respective MS acquisition modes used. The MS details and data searching approaches have been described previously.

### 2.18 Antibodies and western blotting

The following antibodies were purchased from Cell Signaling Technology: LC3B (cat. 2775s), CDK12 (cat. 11973s), ERBB2 (cat. 2165s), Phospho‐ERBB2 (Tyr877) (cat. 2241s), FGFR2 (cat. 11835s), Phospho‐FGFR (Tyr653/654) (cat. 3471s), Phospho‐Src Family (Tyr416) (cat. 2101s), Phospho‐mTOR (Ser2448) (cat. 5536), Phospho‐Akt (Ser473) (cat. 9271), Phospho‐MEK1/2 (Ser217/221) (cat. 9154s), and GAPDH (cat. 2118s). β‐actin (cat. sc‐69879) and pan 14‐3‐3 (cat. sc‐1657) were purchased from Santa Cruz Biotechnology (Dallas, TX, USA). HRP‐conjugated secondary antibodies against rabbit (cat. 1706515) and mouse (cat. 1706516) IgG were purchased from Bio‐Rad (Hercules, CA, USA). Standard western blots were undertaken using RIPA lysates as previously described [41].

### 2.19 Cell assays

The MTS (3‐(4,5‐dimethylthiazol‐2‐yl)‐5‐(3‐carboxymethoxyphenyl)‐2‐(4‐sulfophenyl)‐2H‐tetrazolium) assay was used to assess cell proliferation *in vitro* after siRNA or drug treatment. At 96 h, 20 μL of MTS solution was added per well (96‐well plate) and incubated at 37 °C for 30 ~ 60 min. The absorbance was measured at 490 nm using the PheraStar plate reader (BMG Labtech, Ortenberg, Germany). Blank control well measurements were subtracted as background values for each sample well. siRNA knockdown time course data was normalized to day 1 measurements and drug titration data from drug inhibition experiments was normalized to the DMSO negative control values. For colony formation assays, cells were then fixed with methanol and colonies stained with 0.4% (w/v) crystal violet (Sigma). Colony number and colony size were quantified with imagej (Version 1.53a, the laboratory for optical and computational instrumentation, University of Wisconsin, Madison, WI, USA).

### 2.20 Statistical analysis and data visualization

Quantitative data were presented as mean ± standard deviation of the mean (SD). Comparisons between two means were evaluated for differences using two‐sided student's *t*‐test, one‐way ANOVA or two‐sided Wilcoxon test as appropriate. The IC50 outlier values were determined by the Grubbs' data outlier test. To visualize data, a series of R packages were applied, including ggplot2, complexheatmap [42], and ggradar. The differentially regulated proteins and phosphosites between integrative subgroups from three datasets were identified under the thresholds of |log2FC| > 1 and adj.*P* value < 0.05, and mapped to the KEGG pathway network via cytoscape (version 3.9.1) [43]. Survival analysis was applied via the R package *survival*.

For statistical analyses of large‐scale omics data, an FDR‐corrected *P*‐value (adjust *P*) < 0.05 was adopted as statistically significant in the most cases, but some other thresholds were also considered according to the recommendation of the specific high‐throughput statistical methods, such as *P*‐value < 0.05 was adopted in WGCNA and FDR < 0.25 was adopted in gsea. For all statistical analyses of cell assays and clinical data, a *P* value < 0.05 was considered as statistically significant.

## 3 Results

### 3.1 Proteomic landscape of GC cell lines

**Fig. 1:**
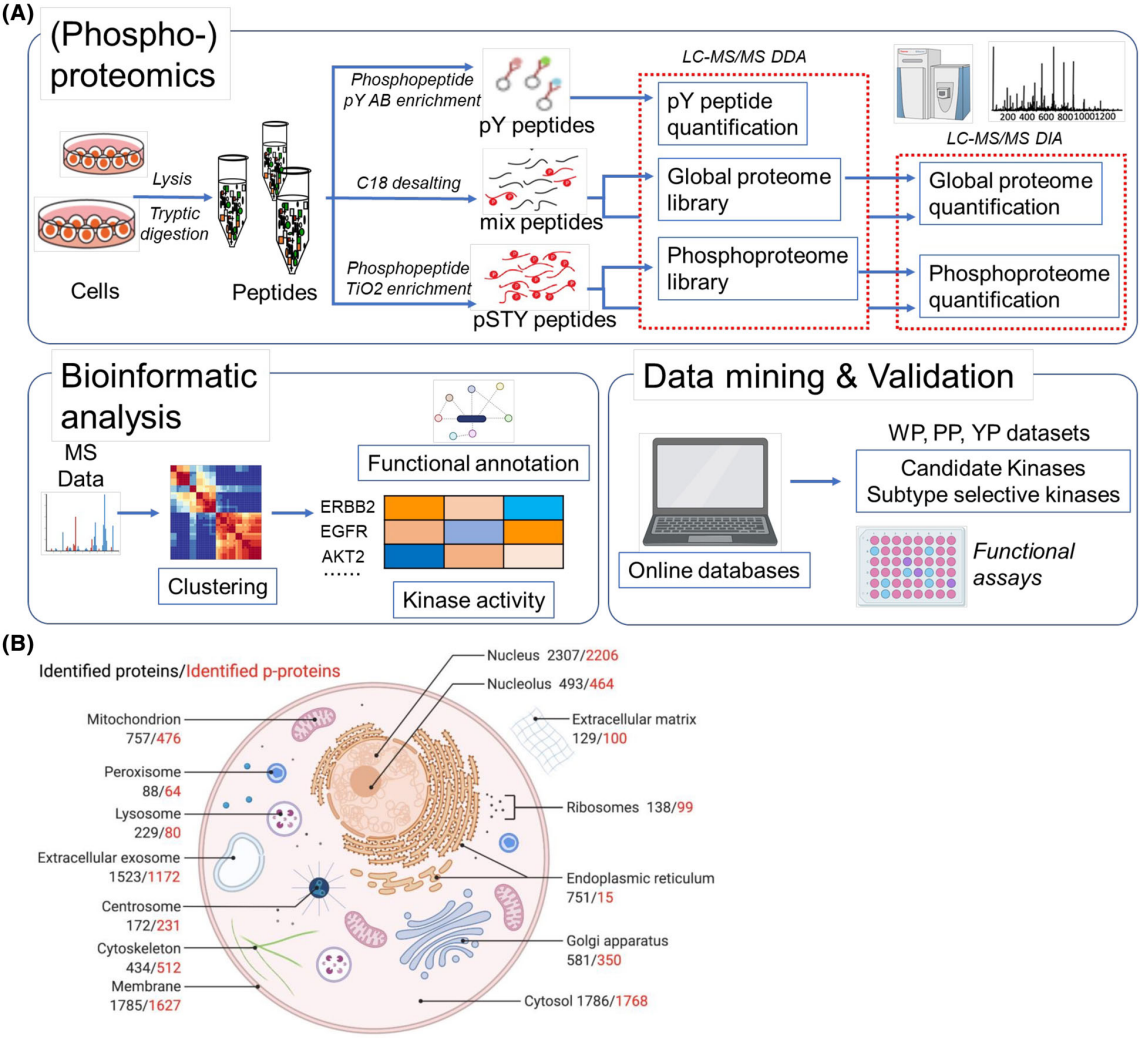
Workflow and cellular landscape of (phospho)proteomics. (A) Schematic of the integrated workflow of (phospho)proteomics, bioinformatic analysis and data mining and validation. WP and phosphoproteome (PP) analyses were conducted on three biological replicates, while tyrosine phosphorylation profiling (YP) was carried out on two biological replicates. Subsequent bioinformatic analyses described in the manuscript utilize these datasets. (B) Subcellular distribution of (phospho)proteins annotated with Gene Ontology. DIA, data‐independent acquisition; LC–MS, liquid chromatography‐mass spectrometry; MS, mass spectrometry; P‐proteins, phosphoproteins.

A panel of 14 GC cell lines (Table S1) was utilized for WP, phosphoproteome (PP) and tyrosine phosphorylation (YP) profiling, followed by downstream bioinformatic and functional analyses (Fig. 1A). This identified 6091 proteins, 16 685 unique phosphopeptides and 8705 phosphorylated Tyr sites across the panel (Fig. S1A,B, Table S3). Correlation analyses revealed strong consistency between the biological replicates for each GC cell line (Fig. S1C). The localization of annotated (phospho)proteins was widely distributed across different cellular organelles, as well as extracellular matrix (Fig. 1B), consistent with the unbiased enrichment and profiling approaches adopted in this study. Taken together, these data sets provide comprehensive coverage of the GC proteome and lay a strong foundation for downstream bioinformatic, validation, and functional analyses.

### 3.2 Subclassification and pathway analysis based on the GC proteome

**Fig. 2:**
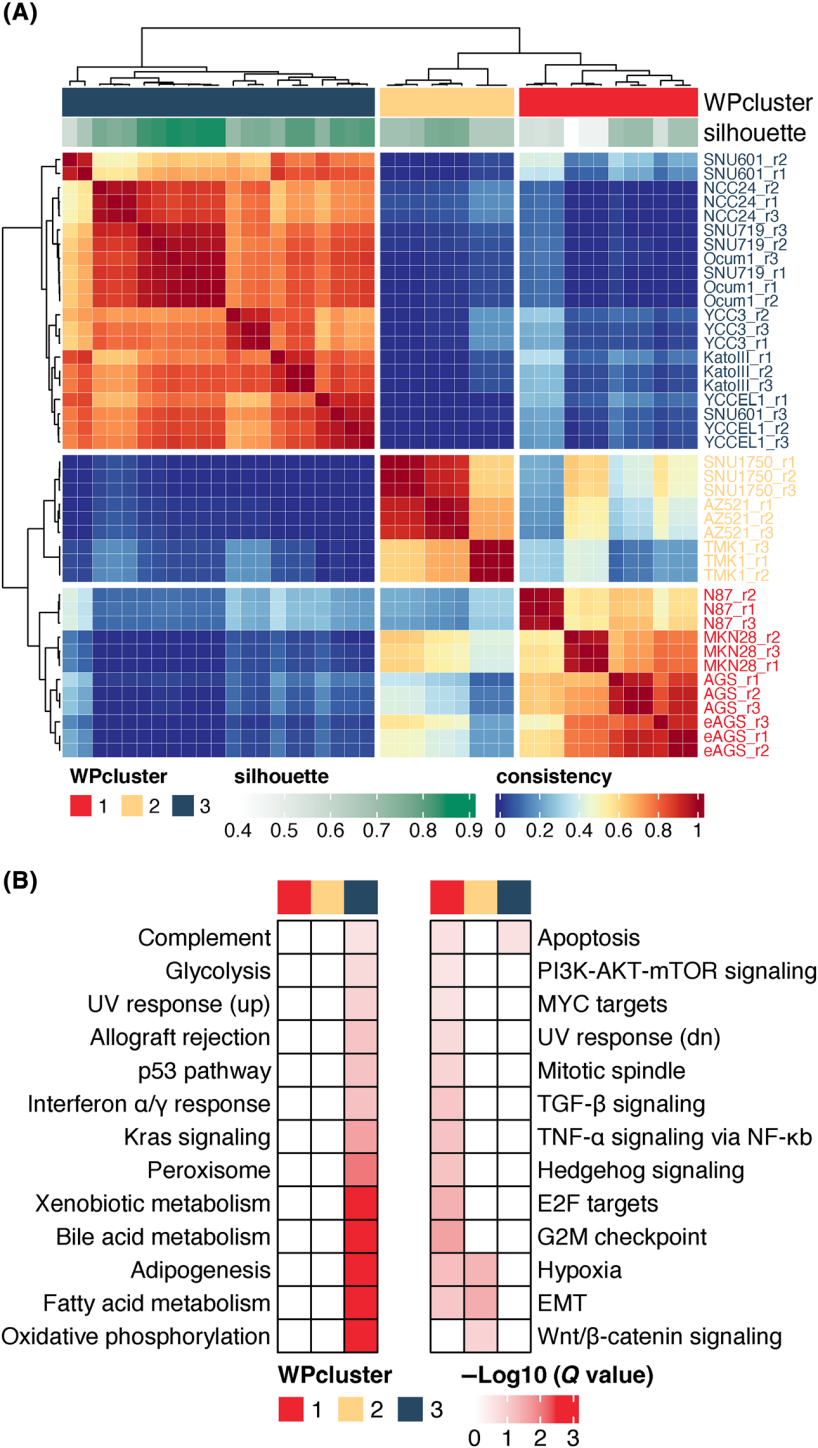
Subclassification based on the proteome and functional annotation of identified subgroups. (A) Non‐negative matrix factorization (NMF) classification using the whole proteome (WP) of gastric cancer (GC) cell lines. The heatmap presents the sample consensus obtained from NMF clustering. The blue‐to‐red gradient denotes the percentage of consistency in the clustering results. The subclassification of basis matrix and consensus matrix had a high consistency and were plotted in different colors. The silhouette score of each sample (which indicates its selective association with a particular subgroup) after 100 times of NMF clustering is indicated with a green‐white gradient. (B) Gene set enrichment analysis (gsea) for particular subclusters identified using the WP dataset. dn, down; EMT, epithelial‐mesenchyme transition; WP, whole proteome.

In order to subclassify the GC cell lines based on their WP, we applied an unsupervised clustering approach termed non‐negative matrix factorization (NMF), resulting in definition of three subclusters (Fig. 2A and Fig. S2A,B). Gene set enrichment analysis (GSEA) was further applied to determine the association of particular biological functions with specific WP subclusters, highlighting that WP cluster 1 was significantly enriched in EMT and several proliferation related pathways, whereas WP cluster 3 was characterized with the enrichment of KRAS and p53 signaling, and many metabolic and immune pathways. Furthermore, hypoxia, WNT signaling, and EMT processes were significantly enriched in WP cluster 2 (Fig. 2B).

Discovering clusters of proteins with high expression correlation can reveal shared biological processes or common regulatory mechanisms [44]. Weighted correlation network analysis (WGCNA) was applied to explore such correlated proteins within the WP subclusters 1–3. As a result, proteins were assigned into 12 modules by WGCNA and the relationship of these modules with particular subclusters and GO enrichment categories determined. The resulting data (Fig. S2C) were consistent with the previous GSEA (Fig. 2B). For example, the pink module, which was enriched for cell migration, and the brown module, which was characterized by mitotic cell cycle, exhibited increased expression in the WP cluster 1. In addition, tight junction assembly and a variety of metabolic processes were found to be associated with the turquoise module, which was positively correlated with the WP cluster 3. To further interrogate subset‐specific proteins, differentially expressed proteins were retrieved by one way ANOVA. Apart from the proliferation‐related terms and metabolic processes differentially enriched across WP clusters, several novel functions in each proteome cluster were only annotated after the ANOVA analysis. For example, snRNA 3′‐end processing was associated with WP cluster 2, while negative regulation of response to DNA damage was associated with cluster 1 (Fig. S2D).

Taken together, the results obtained from different gene filtering approaches and functional annotation methods provide insights into the different biological features associated with the three proteome subsets, with the association of EMT and proliferation‐related processes with cluster 1, and metabolism‐related ones with cluster 3, particularly evident.

### 3.3 Comparison of the WP GC subclassification with other published omics‐based taxonomies

To compare our WP subclassification with other GC subclassifications, we first identified 13–15 featured characteristic proteins as a ‘metagene’ for each WP subtype using factorization approach from NMF (Fig. S3A,B). Then, a prediction model based on these 42 contributing proteins was trained by the machine learning method Neural Networks, and finally applied to assign published GC cohorts, into WP cluster 1–3, designated Cell proliferation/EMT, EMT and Metabolism, respectively. The cohorts assigned comprised a cell line panel (Singapore‐37 [45], *N* = 37 GC cell lines) and clinical cohorts (Singapore cohort [10], *N* = 200; TCGA cohort [8], *N* = 240; ACRG cohort [12], *N* = 300; KUGH cohort [11], *N* = 93; PUCH cohort [14], *N* = 84). All of these datasets were measured at the transcriptomic level, except for the PUCH cohort which was profiled based on the proteome.

**Fig. 3:**
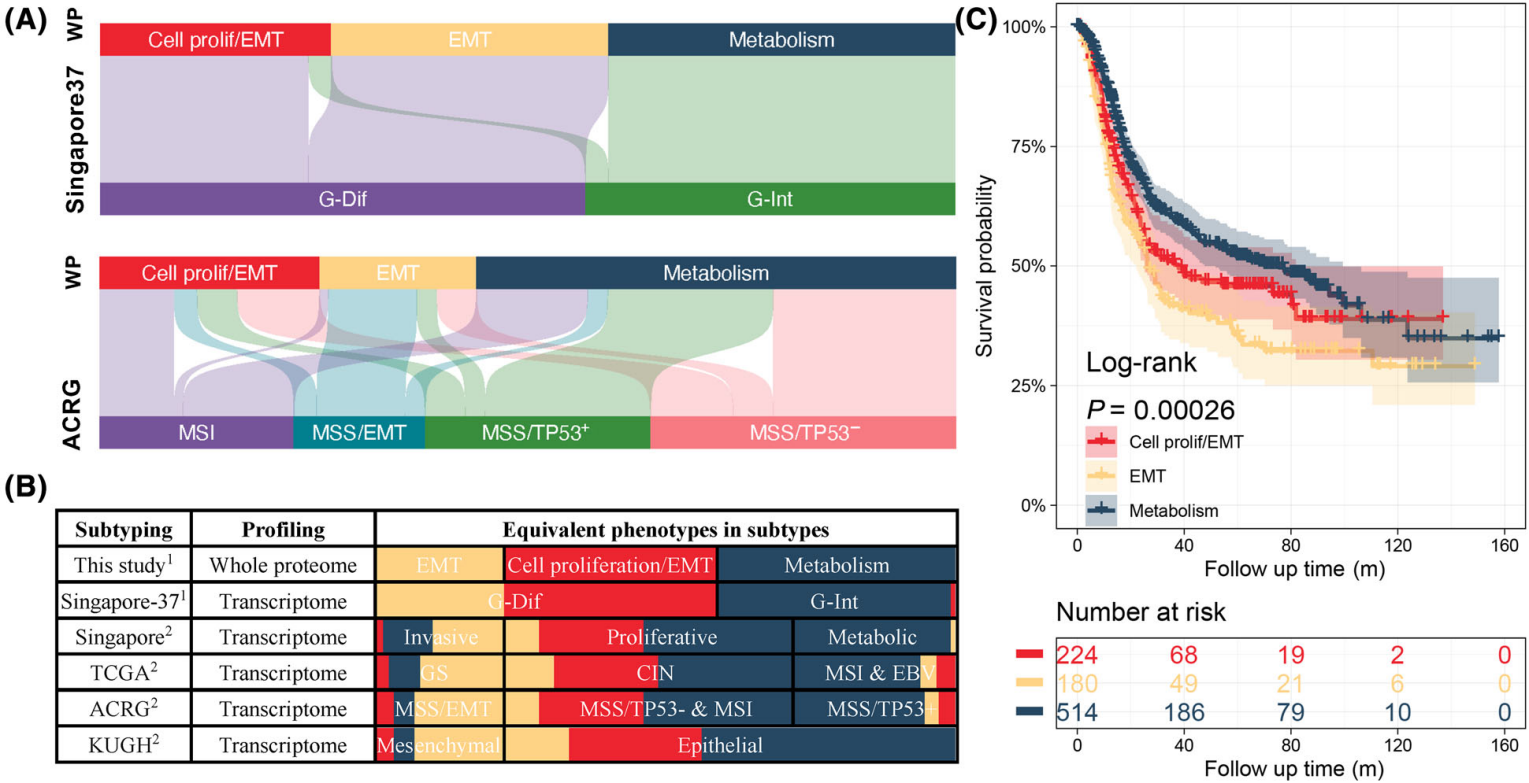
Comparison of whole proteome subclassification with those of published cohorts. (A) Comparison of our whole proteome (WP) subclassification with published Singapore 37‐cell line subtyping and Asian Cancer Research Group (ACRG) clustering. (B) Summary comparison of WP subclassification with gastric cancer (GC) molecular subtypes from different studies. ^1^Based on GC cell lines; ^2^Based on patient tissues. The proportion of each WP cluster in the other subtypes is indicated by the distribution of different colors. Red: cell proliferation/EMT; Yellow: EMT; Blue: Metabolism. (C) Kaplan–Meier survival analysis of published cohorts classified by WP subtyping. All GC patients in Singapore, ACRG, The Cancer Genome Atlas (TCGA) and Kosin University Gospel Hospital (KUGH) cohorts were integrated and reassigned according to WP subclassification to compare the prognostic difference between WP clusters. CIN, chromosomal instability; EBV, Epstein–Barr virus‐positive; EMT, epithelial‐mesenchyme transition; G‐Dif, genomic diffuse; G‐Int, genomic intestinal; GS, genomically stable; MSI, microsatellite instability; MSS, microsatellite stability.

A significant relationship was observed between the WP subclassification and Singapore 37‐cell line clustering, as all genomic diffuse (G‐Dif) GC cell lines were assigned into the Cell proliferation/EMT and EMT cluster, while 93.75% (15/16) of genomic intestine (G‐Int) GC cell lines associated with the metabolism cluster. These assignments are detailed in Table S4, presented schematically in Fig. 3A,B and analyzed statistically in Table S5 (*P* = 7.77 × 10^−10^). Comparison of the WP and ACRG subclusters revealed that 67.39% (31/46) of MSS/EMT patients corresponded to the EMT cluster, whereas 73.42% (58/79) of MSS/TP53+ patients associated with the metabolism cluster, contributing to a highly significant statistical correlation between WP subclassification and ACRG subtyping (*P* = 4.6 × 10^−19^) (Tables S4 and S5, Fig. 3A,B). Furthermore, when the ACRG cohort was subjected to our WP‐based subclassification and the resulting subgroups subjected to gsea at the level of the whole transcriptome, there was high consistency between enriched pathways in corresponding cell line and patient subgroups (Table S6).

Significant relationships were also detected between the WP subclassification and TCGA cohort (*P* = 1.6 × 10^−7^), KUGH cohort (*P* = 1 × 10^−6^), as well as Singapore cohort (*P* = 2.9 × 10^−15^) (Tables S4 and S5, Fig. S3C–E, Fig. 3B). Particularly prominent were correspondence between the majority of the TCGA EBV and MSI subgroups and the WP metabolism one, KUGH MP and WP EMT, and Singapore metabolic and WP metabolism. Furthermore, upon pooling GC patients from the TCGA, ACRG, KUGH, and Singapore cohorts into corresponding WP subclusters, GC patients with EMT‐and metabolism‐subtype tumors had the worst and best prognoses respectively (log rank test, *P* = 3 × 10^−4^) (Fig. 3C), consistent with published findings [12]. Interestingly, the only comparison which did not reach statistical significance involved the PUCH cohort (Tables S4 and S5, Fig. S3F). While this was a proteomic study, the lack of concordance may reflect only diffuse‐type GC being included in this cohort [14]. Overall, these data highlight the close relationship between our cell line proteomic and patient tumor transcriptomic datasets and highlight particular GC cell lines as representative models for specific GC patient subtypes.

### 3.4 Subclassification and molecular features of the GC cell line panel based on the phosphoproteome

**Fig. 4:**
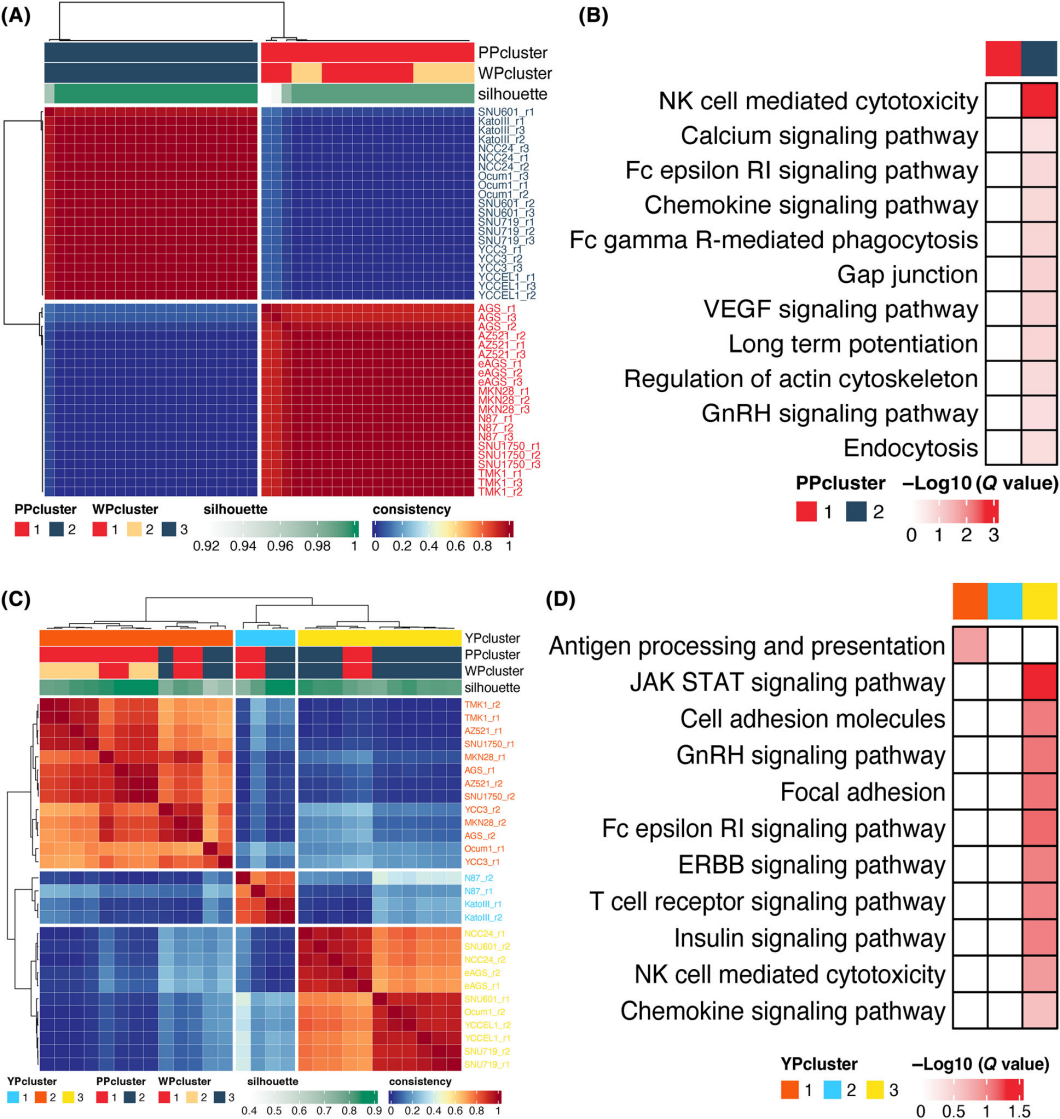
Subclassification based on phosphoproteomics and tyrosine phosphorylation and functional annotations of each subcluster. (A) Non‐negative matrix factorization (NMF) classification using phosphoproteomic (PP) data from gastric cancer (GC) cell lines. Blue‐to‐red gradient denotes the percentage of consensus in the clustering results and the silhouette score of each sample after 100 times of NMF clustering is indicated with a green‐white gradient. (B) Enrichment of Kyoto Encyclopedia of Genes and Genomes (KEGG) gene sets for the different PP subclusters. (C) NMF classification from tyrosine phosphorylation (YP) profiling of GC cell lines. Blue‐to‐red gradient denoted the percentage of consensus in the clustering results and the silhouette score of each sample after 100 times of NMF clustering is indicated with a green‐white gradient. (D) Enrichment of KEGG gene sets for the YP subclusters. Fc epsilon RI, high‐affinity receptor I for the Fc region of immunoglobulin E; NK, natural killer; WP, whole proteome.

Unsupervised clustering of the PP dataset resulted in a binary classification for the GC cell lines, which forged the cell lines in WP clusters 1 and 2 together as PP cluster 1, while WP cluster 3 corresponded to PP cluster 2 (Fig. 4A and Fig. S4A,B). Consistent with WP data (Fig. 2B and Fig. S2C,D), gsea revealed enrichment of immune pathways in PP cluster 2 (Fig. 4B). In addition, the calcium and VEGF signaling pathways were also enriched in this subgroup. To develop this analysis further, statistically different phosphosites were compared between PP clusters. In line with the WP data (Fig. S2C), EMT regulation‐ and cell growth‐related biological processes emerged again in PP cluster 1, and PP cluster 2 exhibited an enrichment for cell–cell junctions (Fig. S4C). WGCNA identified five modules with contrasting associations with the two subclusters (Fig. S4D) and with functional annotations consistent with WP analysis (Fig. 2B and Fig. S2C,D). For example, the black module was positively correlated with PP cluster 1 and regulated the cell cycle, while the high correlation modules of PP cluster 2, for example the pink and green modules, were characterized by cell junction organization, epithelial cell–cell adhesion and Fc‐γ receptor pathway. Interestingly, no metabolism pathway was mapped to PP cluster 2 by any of the functional annotation methods used although it was a marked characteristic for this subset at the proteome level.

A ternary subclassification was obtained upon unsupervised clustering of the tyrosine phosphorylation data, although this was slightly different from that derived from the WP (Fig. 4C and Fig. S4E,F). GSEA revealed that YP cluster 3, which contained many of the cell lines belonging to WP cluster 3 (Metabolism cluster) and PP cluster 2, was associated with particular signaling pathways, including JAK/STAT, GnRH, ERBB, and insulin (Fig. 4D). Furthermore, all of the Tyr phosphosites exhibiting cluster‐selective differences (determined by one‐way ANOVA) were retrieved for functional annotation. In line with gsea, which detected enrichment of receptor signaling pathways in YP cluster 3, this cluster also exhibited an association with the MAPK cascade (Fig. S4G). These analyses highlight how tyrosine phosphorylation profiling complements the other two proteomic screening platforms through its enhanced ability to detect regulation of specific signaling pathways.

Overall, our analysis of the WP, PP, and YP datasets revealed that EMT‐ and cell proliferation‐related processes characterized one or two subgroups of the GC cell line panel, while epithelial processes, metabolic pathways, and the immune response dominated the features of the other subtypes. The analysis also revealed specific signaling pathways (eg RAS, JAK–STAT) that were associated with particular subgroups and represent candidates for pharmacological intervention.

### 3.5 Integrative subclassification based on the three (phospho)proteomic datasets

**Fig. 5:**
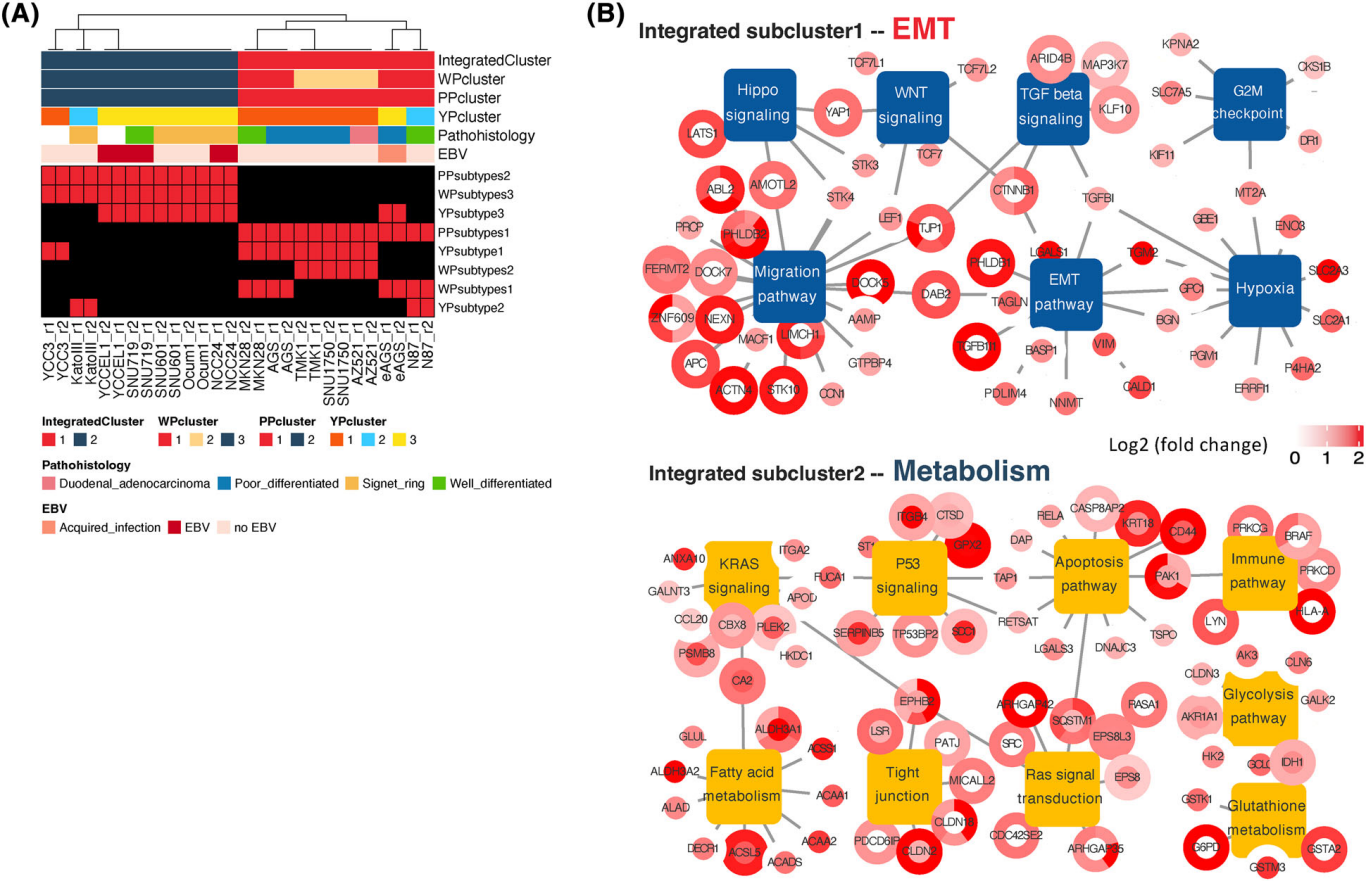
Integrative subclassification and signaling networks of (phospho)proteome‐derived subtypes. (A) Integrative clustering of the three datasets from gastric cancer (GC) cell lines. The *X*‐axis lists 14 GC cell lines, each with two biological replicates, and the *Y*‐axis displays the subtypes identified from clustering of the three datasets. Value 1 (red) indicates that a particular GC cell line was associated with a specific subtype while value 0 (black) indicates the lack of association with any of the identified subtypes. Clinicopathologic characteristics of the cell lines and subclassification from each dataset are also shown. To align with the tyrosine phosphorylation (YP) matrix, only two biological replicates for each cell line from the whole proteome (WP)/phosphoproteome (PP) matrices were included. (B) Integrative network analysis applied to clustering of GC cell lines. The rectangles in navy blue or yellow represent the different gene ontology (GO) terms. Proteins are denoted by double‐layer circles. For each protein, the inner circle represents protein level while the outer circle represents the phosphorylation level of different phosphosites. The color gradient from red to white represents the fold change in the abundance of a given protein or phosphosite. EBV, Epstein–Barr virus‐positive; EMT, epithelial‐mesenchyme transition.

In order to integrate the results of the WP, PP, and YP profiling, a comprehensive clustering approach firstly integrated and transformed the subclassifications into a binary table, and then allocated the cell line panel into two subclusters: S1: AGS, eAGS, AZ521, N87, MNK28, SNU1750, TMK1; S2: KatoIII, NCC24, Ocum1, SNU601, SNU719, YCC3, YCCEL1 (Fig. 5A).

Statistically significant proteomic and phosphoproteomic alterations were then mapped to the GO database to construct integrated networks for each subcluster (Fig. 5B). The network model for subcluster 1 included nodes centred on the EMT, Migration, Wnt, and TGF‐β pathways, with some proteins in these pathways increased at the protein expression level (e.g. AAMP, CCN1, LGALS1, TGFBI, and STK3/4) phosphorylation level (e.g. TGFB1I1, CTNNB1, YAP1, ABL2, and APC) or both (e.g. PHLDB2). Hippo signaling, G2‐M checkpoint and hypoxia were also characteristic of this subcluster. Reflecting the enriched pathways, this integrative subgrouping was named the EMT subcluster (Fig. 5B). Subcluster 2 was characterized by metabolic pathways, such as fatty acid, glycolysis and glutathione metabolism, with proteins upregulated at the expression (e.g. GLUL, ALAD, ACAA1/2, GCLC, and AK3), phosphorylation (e.g. G6PD and GSTA2) or both expression and phosphorylation (e.g. ALDH3A1, ACSL5, and AKR1A1) levels. Consequently, this subgrouping was termed the metabolism subcluster. In addition, RAS and p53 signaling and the apoptosis, immune and tight junction pathways were also enriched in this subcluster (Fig. 5B).

To identify the functions of subcluster‐specific phosphorylation, all the functionally annotated phosphosites from the PhosphoSitePlus database were retrieved and their phosphorylation levels were compared between the two integrative subsets. Interestingly, two opposing processes [46], apoptosis and autophagy, demonstrated contrasting regulation between the two subclusters: phosphosites involved in apoptosis inhibition and autophagy induction were upregulated in the EMT subcluster, while apoptosis inducing sites and autophagy inhibiting sites were highly phosphorylated in the metabolism one (Fig. S5A). These findings were validated by western blotting, which demonstrated that levels of Rb pT821 were higher in the EMT subgroup (Fig. S5B) as originally indicated by our MS analysis. To assess autophagy, we utilized immunoblotting to characterize LC3‐I to LC3‐II conversion in starved cells in the presence of bafilomycin A1, which blocks LC3‐II degradation. This demonstrated markedly increased levels of LC3‐II, and hence autophagy, in the EMT cell lines (Fig. S5C).

Furthermore, phosphorylation associated with increased cell proliferation, DNA repair and motility, and decreased differentiation were in general enhanced in the EMT subcluster, while phosphorylation associated with reduced growth was increased in the metabolism one. In addition, application of a phosphorylation site‐centric functional enrichment analysis approach, PTM‐SEA [34], to the PP data indicated that CDK1‐2 and 4‐6 signaling was enriched in the EMT subset, consistent with the association of this subset with cell proliferation (Fig. S5D).

### 3.6 The kinomic landscape of the GC cell line panel

**Fig. 6:**
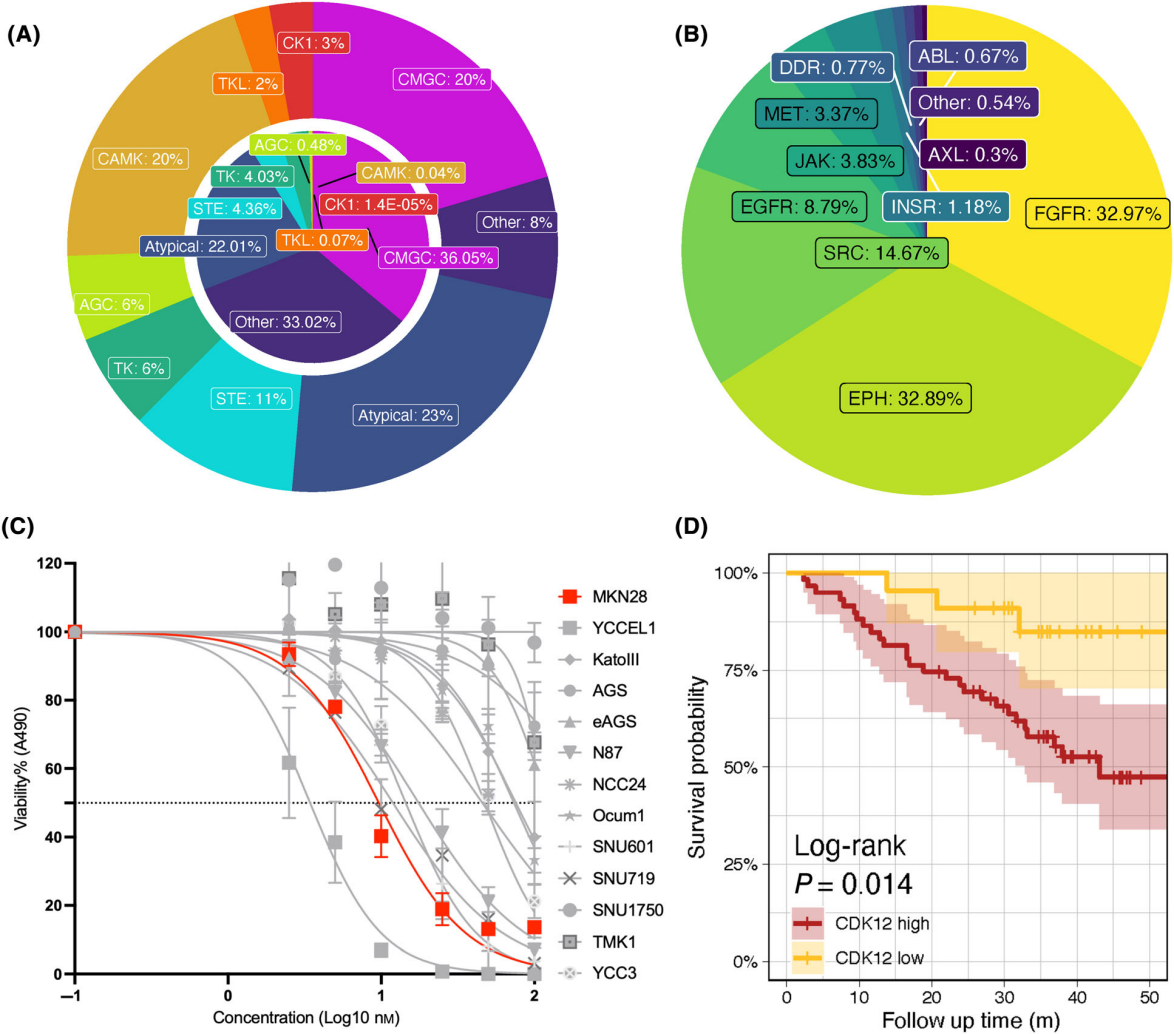
Kinomic landscape and potential oncogenic kinases identified from the gastric cancer cell line panel. (A) Abundance of particular kinase families in the whole proteome (WP) (inner pie chart) and phosphoproteome (PP) (outer circle) datasets. (B) Abundance of kinase families in the tyrosine phosphorylation (YP) dataset. (C) Effects of SR3029 on cell viability of the 13 gastric cancer (GC) cell lines. Data from a MTS indirect cell viability/proliferation assay at Day 4 are expressed relative to DMSO control, which was arbitrarily set at 1. Error bars represent the standard deviation of the mean from *n* = 3 independent experiments. (D) Kaplan–Meier survival analysis based on predicted activity of a specific outlier kinase (CDK12).

After searching against the UniProt and PhosphoSitePlus databases, 198, 193, and 140 kinases were mapped to the WP, PP, and PY datasets, respectively. The proportions of peptide abundance from each kinase family in each dataset are presented in Fig. 6A,B. Peptides from the CMGC, CAMK and atypical kinase families made the largest individual contributions to the kinome proteomic data, comprising over 60% of peptide intensity. Over one third of kinome PP intensity (36%) was derived from the CMGC family, while nearly two thirds of kinome PY intensity was contributed by the FGFR and EPH kinase families.

In order to identify the ‘outlier’ kinases with extreme expression/activation in cell line subsets that may represent kinase oncogenic ‘drivers’ and hence therapeutic targets, the top 5% of molecular features with the highest expression/phosphorylation level across all samples were defined. As a result, 124 outlier kinases were identified from the three datasets. For instance, as indicated by the WP and PP heatmaps, ERBB2 and CDK12 proteins were overexpressed and hyperphosphorylated on specific serine/threonine residues in N87 cells (Fig. S6A,B), whereas based on the YP and PP heatmaps, multiple tyrosine and serine residues of FGFR2 were highly phosphorylated in KatoIII cells (Fig. S6B,C). Increased expression of CDK12 and ERBB2 in N87 cells, and FGFR2 in KatoIII cells, were confirmed by western blotting (Fig. S6D). This approach also confirmed elevated ERBB2 pY877 and FGFR pY653/654 (activation loop residues conserved across the FGFR family) in N87 and KatoIII cells, respectively.

To characterize the dependency of specific cell lines on these outliers, we utilized siRNA directed against ERBB2 and FGFR2, as well as lapatinib (an ERBB2 inhibitor). Targeting FGFR2 and ERBB2 strongly inhibited proliferation of KatoIII and N87 cells, respectively, validating these outlier kinases as therapeutic targets, consistent with published data (Fig. S6E) [47, 48]. Furthermore, since MKN28 was characterized by high phosphorylation of CSNK1D_S384 and CSNK1E_S389 (Fig. S6B), we tested SR3029, a CSNK1D/E inhibitor that has not been evaluated in GC. This revealed that MKN28 cells exhibited high sensitivity to SR3029, second only to the YCCEL1 line (YCCEL1 vs MKN28, *P* = 2.5 × 10^−3^; MKN28 vs. SNU719, *P* = 3.9 × 10^−2^) (Fig. 6C). Due to the lack of availability of appropriate CSNK1D/E phosphospecific antibodies, we confirmed on‐target activity of SR3029 by MS‐based phosphoproteomic analysis, which revealed marked inhibition of CSNK1D S384 phosphorylation by drug treatment (Fig. S6F). Given that CDK4 and CDK6 are key off‐target kinases of SR‐3029 [49] and both of them are significantly more highly expressed in YCCEL1 than MKN28 cells (*P* = 1 × 10^−4^, *P* = 2.14 × 10^−2^, respectively; Student test) (Fig. S6G), the extreme sensitivity of YCCEL1 cells could be associated with this off‐target effect of SR‐3029.

To further explore the clinical relevance of these 124 outlier kinases, 1512 GC tumors from publicly available databases [39] were interrogated for somatic mutation and amplification of outlier kinases, revealing that 81% of the specimens had DNA alterations of the queried kinases. Outliers, numbering 32 in total, exhibited genomic alterations, mainly amplification, in at least 5% of patients, including a number of kinases targetable by FDA‐approved small molecule inhibitors, such as ERBB2, EGFR, and CDK6 (Fig. S6H). Of these 32 outlier kinases, 17 were associated with unfavorable prognosis, including CDK12, SRC, and IGF‐1R, suggesting an association with disease progression (Fig. 6D, Fig. S6I,J, Table S7).

### 3.7 Src family kinases may have broad applicability as therapeutic targets in GC

**Fig. 7:**
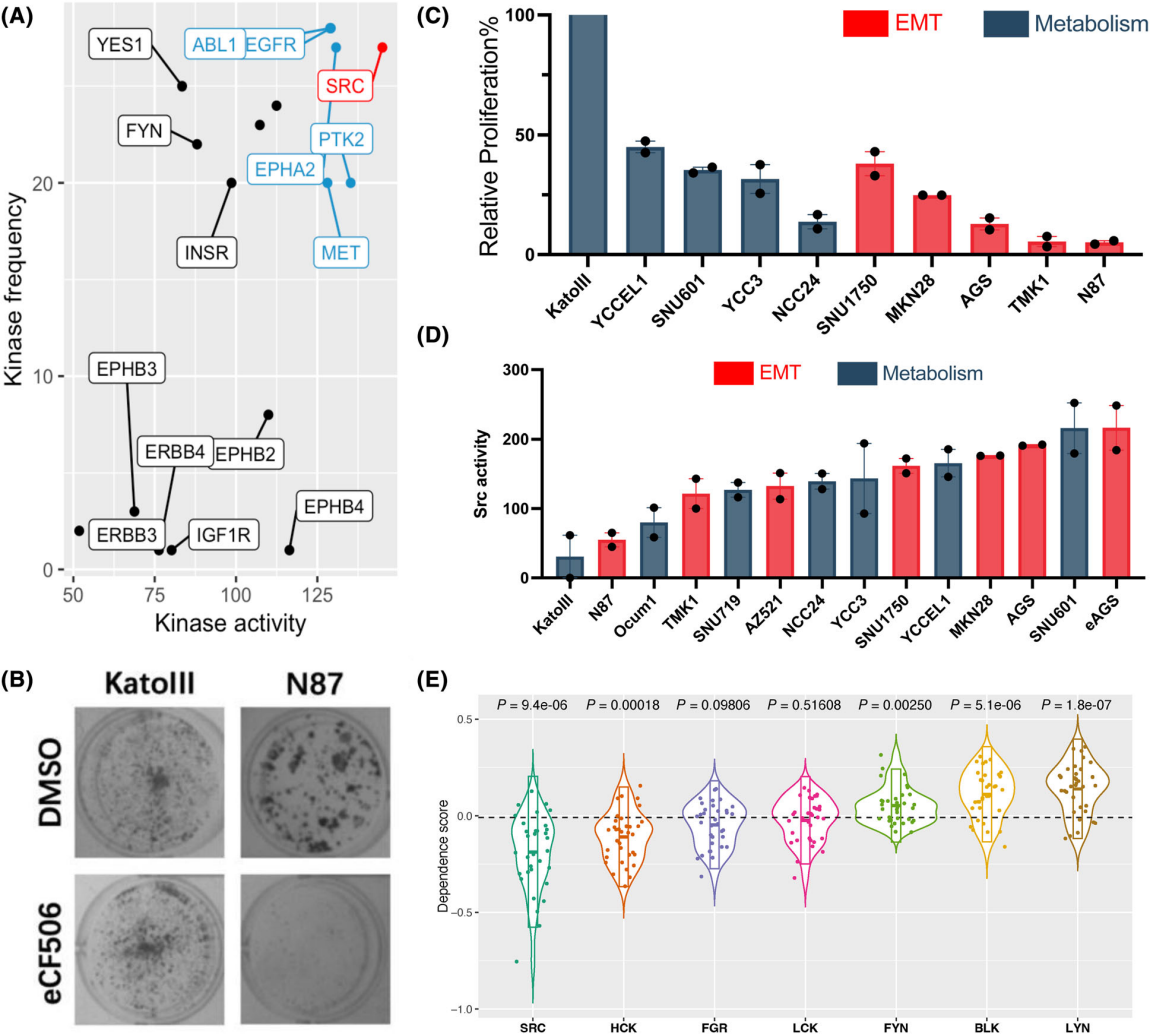
Assessing the vulnerability of the gastric cancer cell line panel to Src Family Kinase Inhibition. (A) The frequency and activity of Tyrosine kinases across the gastric cancer (GC) cell line panel inferred from Integrative Inferred Kinase Activity (INKA) scoring. See also Fig. S7. (B) The effect of the Src family kinase (SFK) inhibitor eCF506 on colony formation of KatoIII and N87 cell lines. (C) Quantification of the colony formation assay across the cell line panel. eCF506 (250 nm) treatment data are expressed relative to vehicle control (DMSO) which was arbitrarily set at 1. Error bars represent the range of the mean from *n* = 2 independent experiments. (D) Predicted Src activity of each GC cell line. This was extracted from the Tyr kinase activity data, with error bars representing the range of the mean from *n* = 2 independent tyrosine phosphorylation profiling experiments. (E) Gene dependency scores of 7 individual SFKs in 34 GC cell lines, extracted from the DepMap database. The boxplot overlaid on the violin plot indicates the mean ± SD. The *P* values indicate results from a student's‐*t* test comparing dependency on a particular SFK to all of the SFKs. EMT, epithelial‐mesenchyme transition.

In the YP dataset, INKA scoring [35] was applied to obtain an optimized value and ranking for inferred kinase activities, defining the top 10 activated tyrosine kinases (Fig. S7A). Taking into consideration the kinase activity and the frequency of assignment as a top 10 kinase across the cell line panel, Src was located in the first tier, followed by five other kinases: ABL1, EGFR, EPHA2, PTK2, and MET (Fig. 7A). To determine whether sensitivity to Src inhibition was consistent with this prediction, the second generation, highly selective Src inhibitor eCF506 [50] was tested in a colony formation assay. This drug strongly inhibited colony formation of all cell lines except KatoIII (Fig. 7B,C). Since both KatoIII and N87 cells exhibit low Src activity (Fig. 7D), but markedly contrasting eCF506 sensitivity (Fig. 7C), the eCF506 resistance of KatoIII may reflect high level FGFR2 amplification in these cells [47]. Of note, in general, Src activation loop phosphorylation as determined by western blotting was consistent with predicted Src activity (Fig. S7B), although Ocum1 demonstrated unexpectedly high Y416 phosphorylation by western blotting. However, it should be noted that the predicted activity takes into account the phosphorylation level of kinase substrates as well as the kinase itself.

Since eCF506 inhibits all Src family kinases (SFKs) [50], GC cell line dependency on individual members of the Src family was mined in Depmap in order to identify SFKs most likely to contribute to eCF506 sensitivity. This indicated that the greatest dependency is on SRC followed by HCK, suggesting that eCF506 sensitivity of GC cell lines predominantly reflects targeting of SRC and HCK in combination (Fig. 7E).

### 3.8 Identifying and targeting kinomic vulnerabilities in the GC cell line subgroups

**Fig. 8:**
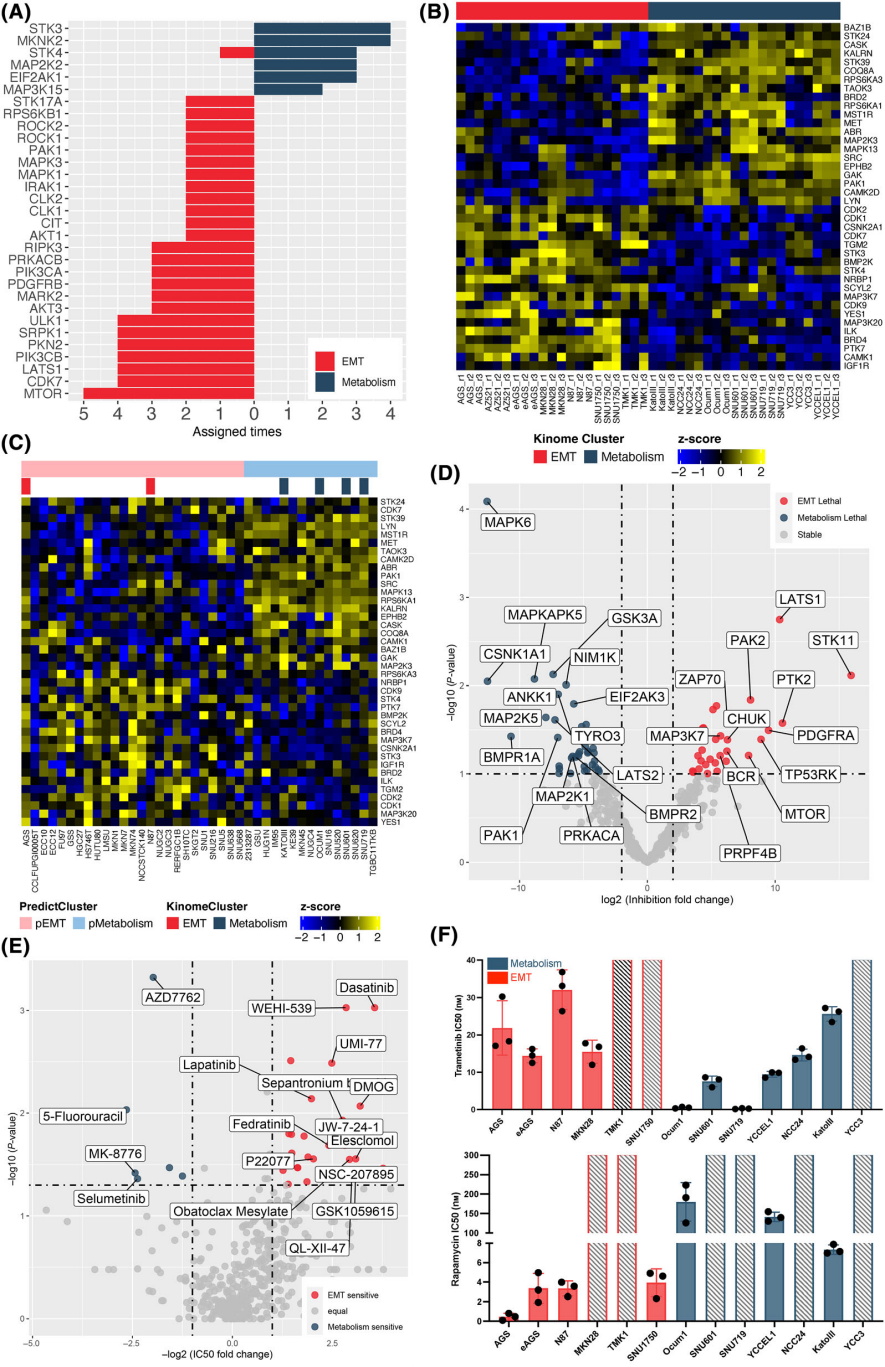
Potential vulnerabilities in subgroups of gastric cancer cell line panel. (A) Scoring summary of each kinase assigned as differentially activated in the epithelial‐mesenchyme transition (EMT) or metabolism subgroups. The number of assignments indicate the number of times a kinase was assigned to one of the two subgroups using the six activity matrices (see Materials and methods). (B) Subclassification of the cell line panel using 40 differentially expressed kinases. Yellow represents high protein abundance, whereas navy blue represents low expression level. (C) Differentially expressed kinases and kinomic subclassification across the Cancer Cell Line Encyclopedia (CCLE) gastric cancer (GC) cell lines. The predicted subclassification of the CCLE GC cell lines is shown at the top with light colors. The distribution of overlapping cell lines from our in‐house analysis are highlighted underneath in red and navy blue, corresponding to EMT and Metabolism, respectively. (D) Volcano plot of subtype‐specific kinase dependencies mined from DepMap. Colors indicate subtype‐selective thresholds of *P* < 0.1 and log2 FC > 2 (red), or *P* < 0.1 and log2 FC < −2 (navy blue). (E) Volcano plot of subtype‐specific drugs. Colors indicate subtype‐selective thresholds of *P* < 0.05 and −log2 FC > 1 (red), or *P* < 0.05 and log2 FC < −1 (navy blue). (F) IC50 values for inhibition of viability of GC cell lines in EMT subgroup (red) and metabolism subgroup (blue). The IC50 value was deduced by dose titration assessment using a MTS indirect cell viability/proliferation assay. The cell lines with IC50 values exceeding the maximum screening concentration are indicated by gray slash shading. Error bars represent the standard error of the mean from *n* = 3 independent experiments. Trametinib treatment (top, *P* = 0.002); Rapamycin treatment (bottom, *P* < 0.001).

Given the PP and integrative subclassifications were consistent (Figs 4A and 5A), we predicted kinase activities based on the PP dataset in order to identify differentially activated kinases in the EMT and metabolism subgroups (see Materials and methods) (Fig. S8A and Fig. 8A). This predicted that MTOR, CDK7, and LATS1 were preferentially activated in the EMT subgroup, and STK3/4, MKNK2, MAP2K2, and EIF2AK1 in the metabolism group. We then set out to cross‐validate these data with orthogonal approaches. However, only a limited number of our cell line panels are present in the CCLE, the latter of which does not contain phosphoproteomic data. Consequently, we utilized an indirect route. First, we extracted kinases from the WP dataset and subjected them to NMF clustering. Interestingly, the resulting kinomic subclassification was highly similar to that generated from the PP dataset and integrative clustering, thereby resolving the EMT and metabolism subgroups (Fig. S8B,C). Furthermore, this segregation of the cell line panel could be recapitulated with a set of 40 differentially expressed kinases (Fig. 8B). Of note, this approach highlighted that in general, predicted kinase activity (Fig. 8A) did not follow kinase expression (Fig. 8B). Second, by utilizing mRNA rather than protein expression, we determined how these kinases segregated the 40 GC cell lines in the CCLE. Importantly, this resulted in two subsets that featured appropriate segregation of cell lines present in our cell line panel (Fig. 8C). This strongly suggested the resolution of GC EMT and metabolism subgroups in the CCLE. We were then in a position to apply orthogonal approaches to the CCLE. Mining the dependency score of the CCLE GC cell lines from the DepMap database revealed that dependency on *MTOR* and *LATS1* characterized the EMT subgroup and on *MAP2K1* and *EIF2AK3* the metabolism group (Fig. 8D), consistent with the kinase activity‐based approach (Fig. 8A). To complement this approach, we retrieved the IC50 data for small molecule drugs from the GDSC database and compared drug sensitivity of the EMT and metabolism subgroups. Consistent with kinase activity assignment (Fig. 8A) and dependency data (Fig. 8D), selumetinib (MAP2K1/2i) sensitivity was associated with the metabolism subgroup, while GSK1059615 (PI3K/MTORi) and JW‐7‐24‐1 (CDK7i) were associated with the EMT group (Fig. 8E), respectively. Of note, relative sensitivity to 5‐flurouracil was associated with the metabolism subgroup, indicating that kinase signatures could be used to stratify GC patients according to chemosensitivity.

To validate these predictions, western blotting was first employed to confirm differential phosphorylation of mTOR_S2448, AKT_S473, and MAP2K1/2_S217/221 between the two subgroups (Fig. S8D). Furthermore, sensitivities to the MAP2K1/MAP2K2 inhibitor trametinib and the mTOR inhibitor rapamycin were evaluated across the cell line panel. Apart from some cell lines that failed to demonstrate effective inhibition at the highest concentration (1000 nm) utilized and were identified as statistical outliers by the Grubbs' test, trametinib was more effective at inhibiting metabolism than EMT subgroup cell lines, while rapamycin exhibited greater efficacy in the EMT subgroup (Fig. 8F). Although a number of kinases in the PI3K–AKT axis (i.e., PIK3CA, PIK3CB, AKT1, AKT3) were also preferentially activated in the EMT subgroup (Fig. 8A), differential sensitivity to a pan‐class I PI3K inhibitor or pan‐AKT inhibitor was not detected (Fig. S8E).

## 4 Discussion

In this study, we have undertaken the most comprehensive (phospho)proteomic analysis of GC cell lines to date, providing novel biological insights into this malignancy and through integration with publicly available patient data, identified corresponding experimental models for certain GC subtypes and highlighted potential general and subtype‐selective therapeutic targets.

Currently, ERBB2 and VEGFR2 exhibit corresponding FDA‐approved therapies in GC [51], although many patients still do not benefit from precision treatment. For example, trastuzumab only targets 7–34% of patients with GC and less than 60% of targeted patients respond [16, 52]. Supporting the potential for additional targeted treatments, aberrations occur in several other kinase oncogenes in this malignancy, including EGFR, mTOR, and MET [8, 53]. In addition, the VIKTORY Umbrella trial indicated that patients with GC can benefit from targeted treatments directed via the use of companion biomarkers [17]. To further develop this approach, we applied an outlier approach to our (phospho)proteomic datasets and integrated this with publicly available data regarding genomic aberrations in patient tumors. Specific GC cell lines exhibited marked overexpression/activation of ERBB2 and FGFR2, consistent with previous studies [47, 48]. However, a novel finding was marked phosphorylation of CSNK1D/E, and this correlated with increased sensitivity to a corresponding small molecule inhibitor. Of note, CSNK1D has been highlighted as a potential therapeutic target in breast cancer [54]. In addition, inhibition of another outlier kinase, CDK12, can boost the effect of other anticancer drugs, including sorafenib as well as PARP and immune checkpoint inhibitors [55]. A further important finding stemming from the interrogation of the tyrosine phosphorylation dataset was the common activation of SFKs in GC, which was consistent with broad sensitivity to the next‐generation, highly‐selective SFK inhibitor eCF506 and built upon a previous study describing sensitivity to the multi‐kinase inhibitor dasatinib in GC [56]. Finally, we also report sensitivity to mTOR and MEK inhibitors in the EMT and Metabolism subgroup cell lines, respectively. Overall, our work highlights several novel approaches for the precision treatment of GC.

Several studies have applied omic approaches to subclassification of GC [8, 9, 10, 11, 12, 13, 14], but an important issue is the identification of cell line models for the identified subtypes to facilitate pre‐clinical research. Importantly, through application of (phospho)proteomics and machine learning we were able to identify cell line models for particular patient‐derived subtypes across various GC subtyping systems. For example, there was a prominent correspondence between the ACRG MSS/EMT, TCGA GS, KUGH mesenchymal, Singapore invasive, and the WP EMT cluster, while the ACRG MSS/TP53^+^, TCGA EBV, and MSI and Singapore metabolic subgroup exhibit similarity to the WP metabolism cluster. Our WP subclassification also highlighted models for the G‐Dif and G‐Int subgroups in the Singapore 37 study, as well as their alignment with the Lauren histopathological classification of GC. Notably, two distinct pathways, namely, EMT and gland development, were enriched in the WP EMT and metabolism clusters, respectively, consistent with diffuse‐type GC being characterized by EMT features and large glandular lumina being typical of intestinal‐type GC [5, 11]. However, certain subgroups, such as MSI tumor in TCGA, MSS/TP53^−^ subset in ACRG did not clearly align with any of the WP subclusters. These non‐congruences may arise from the ethnic backgrounds of particular samples, a lack of correlation between mRNA and protein, differences in omics profiling techniques, and the use of homogenous cell lines versus complex and heterogeneous tumor tissues.

Although the tumor microenvironment (TME) and tumor‐infiltrating lymphocytes have been extensively studied in GC, an independent immune subtype in GC has only been proposed from molecular profiling of bulk tumor tissues recently [9, 14]. However, our identification of an immune‐enriched subtype of GC cell lines indicates that in addition to the TME, cancer cells themselves may also express immune‐related genes, in line with a single‐cell RNA‐seq study reporting that immune mimicry represents a cancer cell hallmark [57]. When the immunomimetic GC cell line subtype (metabolism subtype) is considered in the context of tumor tissue, pathways such as activated chemokine signaling, antigen presentation, and interferon production may lead to a relatively better prognosis, immune cell aggregation and subsequent response to immunotherapy [58, 59, 60]. In contrast, the other GC subsets may be associated with immune suppression due to high expression of TGF‐β and TNF‐α signaling, thereby leading to a poor prognosis in the clinic [59, 61]. Furthermore, the immune subtype of GC also presented a hypermetabolic state, which in the *in vivo* context, may help defend against the immune system via limiting metabolic supply to T cells [62]. Thus, targeting metabolic pathways in the immune‐activated GC cell line subgroup represents a strategy for overcoming the undesired metabolic competition between the tumor and immune system and complement immunotherapy [63].

Enhanced expression and phosphorylation of components of the PI3K‐AKT–mTOR axis represented a molecular characteristic of our EMT GC cell lines and has also been reported for the mesenchymal subtype of GC patients [9, 10]. However, mTOR, and not PI3K or AKT, represented a selective treatment modality against EMT cell lines compared to the metabolism subtype. In the EMT subgroup, highly activated mTOR and PDGFRB, as well as an enriched TGF‐β signaling pathway, indicate that the PDGF‐TGF‐β‐mTORC2 signaling might induce AKT phosphorylation in a PI3K‐independent fashion [64]. This may explain why the PI3K inhibitor lacked potency. In addition, since AKT inhibits FOXO‐mediated RTK expression, the inhibitory effect of the AKT inhibitor in the EMT subgroup could be counteracted by RTK rebound [65].

## 5 Conclusions

By undertaking comprehensive and integrated (phospho)proteomics on a broad GC cell line panel, as well as cross‐validating the data across publicly accessible databases and combining with functional assays, our study provides important insights to further guide subclassification and precision treatment of GC.

## Conflict of interest

The authors declare no conflict of interest.

## Author contributions

CH conducted the experiments, analyzed the results, and wrote the first draft. JS, TK, EVN, and XS were responsible for the methodology and experiment design. JS, TK, EVN, and RJD supervised this study. RJD conceived and designed the study and revised the manuscript. All authors read and approved the final manuscript.

### Peer review

The peer review history for this article is available at https://www.webofscience.com/api/gateway/wos/peer-review/10.1002/1878‐0261.13654.

## Supporting information


**Table S1.** Histopathology and molecular features of 14 gastric cancer cell lines involved in this study.

**Table S2.** The detailed information on DIA properties and inhibitors.

**Table S3.** Processed (phospho)proteome datasets after log2 transformation, normalization, imputation and batch effect adjustment.

**Table S4.** The reassignment of different GC cohorts by WP subclassification.

**Table S5.** A statistical correspondence between WP subclassification and other published omics subclassification of GC.

**Table S6.** GSEA pathway enrichment among three WP subclusters.

**Table S7.** Survival relevance of outlier kinases across multiple GC cohorts.

**Fig. S1.** Phosphoproteomic data overview.
**Fig. S2.** Subclassification based on WP and biological processes associated with each subcluster.
**Fig. S3.** Metagene‐specific proteins and comparison of WP subclassification with other published taxonomies.
**Fig. S4.** Subclassification based on phosphoproteomics and tyrosine phosphorylation and biological processes associated with each subcluster.
**Fig. S5.** Functional annotation of phosphosites and western blotting validation for the integrative subsets.
**Fig. S6.** Outlier kinases in the GC cell line panel and GC patient cohort.
**Fig. S7.** Tyrosine kinase activity in GC cell lines.
**Fig. S8.** Kinomic subclassification and group‐specific vulnerabilities of GC cell line panel.

## Data Availability

The MS proteomics data are available at the ProteomeXchange Consortium under accession code PXD048538. The TCGA validation data used in this study are available in the FIREHOSE database [http://firebrowse.org/?cohort=STAD]. The Singapore 37, ACRG, KUGH, and Singapore expression data used in this study are available in the GEO database under accession codes GSE15460, GSE66229, GSE26899, and GSE15459. The PUCH proteomics data used in this study is accessible in the PRIDE Archive under the accession number PXD008840. The gene expression profiles of GC cell lines associated with drug sensitivity could be accessed at the DepMap data portal (https://depmap.org/portal/). All other data needed to evaluate the conclusions in the paper are present in the article or the Supporting Information. The prediction model and python script are deposited at https://zenodo.org/record/8041616.
