# Supplementary material for: Proteome‐based molecular subtyping and therapeutic target prediction in gastric cancer
Source: Mol Oncol. 2024 Apr 16;18(6):1437–59. doi: 10.1002/1878-0261.13654 (PMC11161736; doi:10.1002/1878-0261.13654)
Supplement: Supplementary file 8 — Fig. S1. Phosphoproteomic data overview. Fig. S2. Subclassification based on WP and biological processes associated with each subcluster. Fig. S3. Metagene‐specific proteins and comparison of WP subclassification with other published taxonomies. Fig. S4. Subclassification based on phosphoproteomics and tyrosine phosphorylation and biological processes associated with each subcluster. Fig. S5. Functional annotation of phosphosites and western blotting validation for the integrative subsets. Fig. S6. Outlier kinases in the GC cell line panel and GC patient cohort. Fig. S7. Tyrosine kinase activity in GC cell lines. Fig. S8. Kinomic subclassification and group‐specific vulnerabilities of GC cell line panel. [file MOL2-18-1437-s003.pdf]

Proteome-based molecular subtyping and therapeutic target prediction in gastric cancer

Changyuan Hu, Jiangning Song, Terry Kwok-Schuelein, Elizabeth V. Nguyen, Xian Shen, Roger J. Daly.

Supplementary Figures

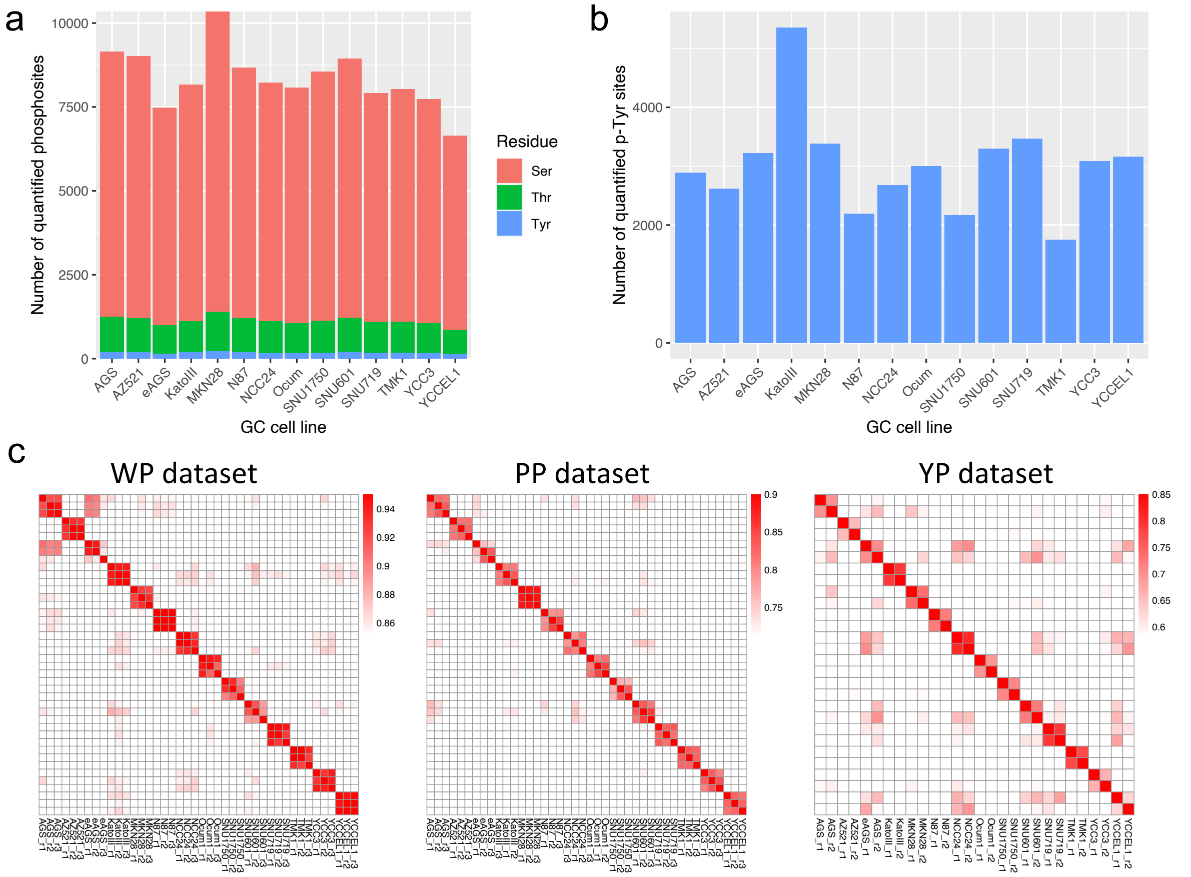

Figure S1. Phosphoproteomic data overview

a Number of Ser/Thr/Tyr phosphosites identified across the cell line panel. **b** Number of Tyr phosphosites across the different GC cell lines. **c** Sample correlation analysis of the WP, PP and YP datasets. Cell lines with biological replicates were plotted together. Triplicates were used for the WP and PP datasets and duplicates for YP. Sample correlation heatmaps highlight the high consistency of the WP, PP and YP data (mean pearson score was 0.93, 0.82 and 0.72, respectively).

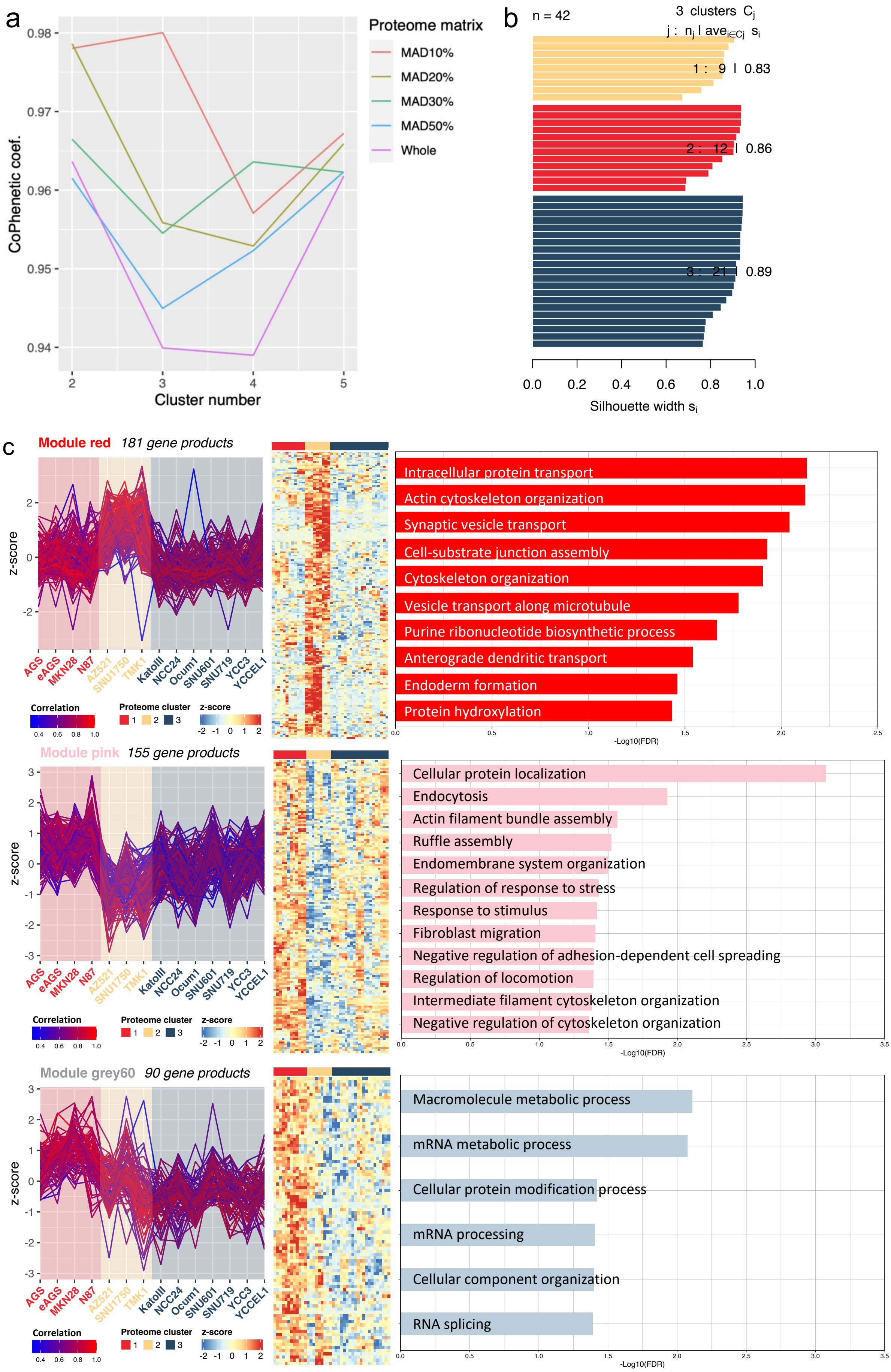
a
Proteome matrix
MAD10%
MAD20%
MAD30%
MAD50%
Whole
CoPhenetic coef.
Cluster number
b
n = 42
3 clusters C_j
j : n_j | $ave_{i \in C_j}$ s_i
1 : 9 | 0.83
2 : 12 | 0.86
3 : 21 | 0.89
Silhouette width s_i
c
Module red 181 gene products
Intracellular protein transport
Actin cytoskeleton organization
Synaptic vesicle transport
Cell-substrate junction assembly
Cytoskeleton organization
Vesicle transport along microtubule
Purine ribonucleotide biosynthetic process
Anterograde dendritic transport
Endoderm formation
Protein hydroxylation
Module pink 155 gene products
Cellular protein localization
Endocytosis
Actin filament bundle assembly
Ruffle assembly
Endomembrane system organization
Regulation of response to stress
Response to stimulus
Fibroblast migration
Negative regulation of adhesion-dependent cell spreading
Regulation of locomotion
Intermediate filament cytoskeleton organization
Negative regulation of cytoskeleton organization
Module grey60 90 gene products
Macromolecule metabolic process
mRNA metabolic process
Cellular protein modification process
mRNA processing
Cellular component organization
RNA splicing
z-score
AGS
eAGS
MKN28
N87
AZ521
SNU1750
TMK1
KatoIII
NCC24
Ocum1
SNU601
SNU719
YCC3
YCCEL1
Correlation
Proteome cluster
-Log10(FDR)

Figure S2c cont.

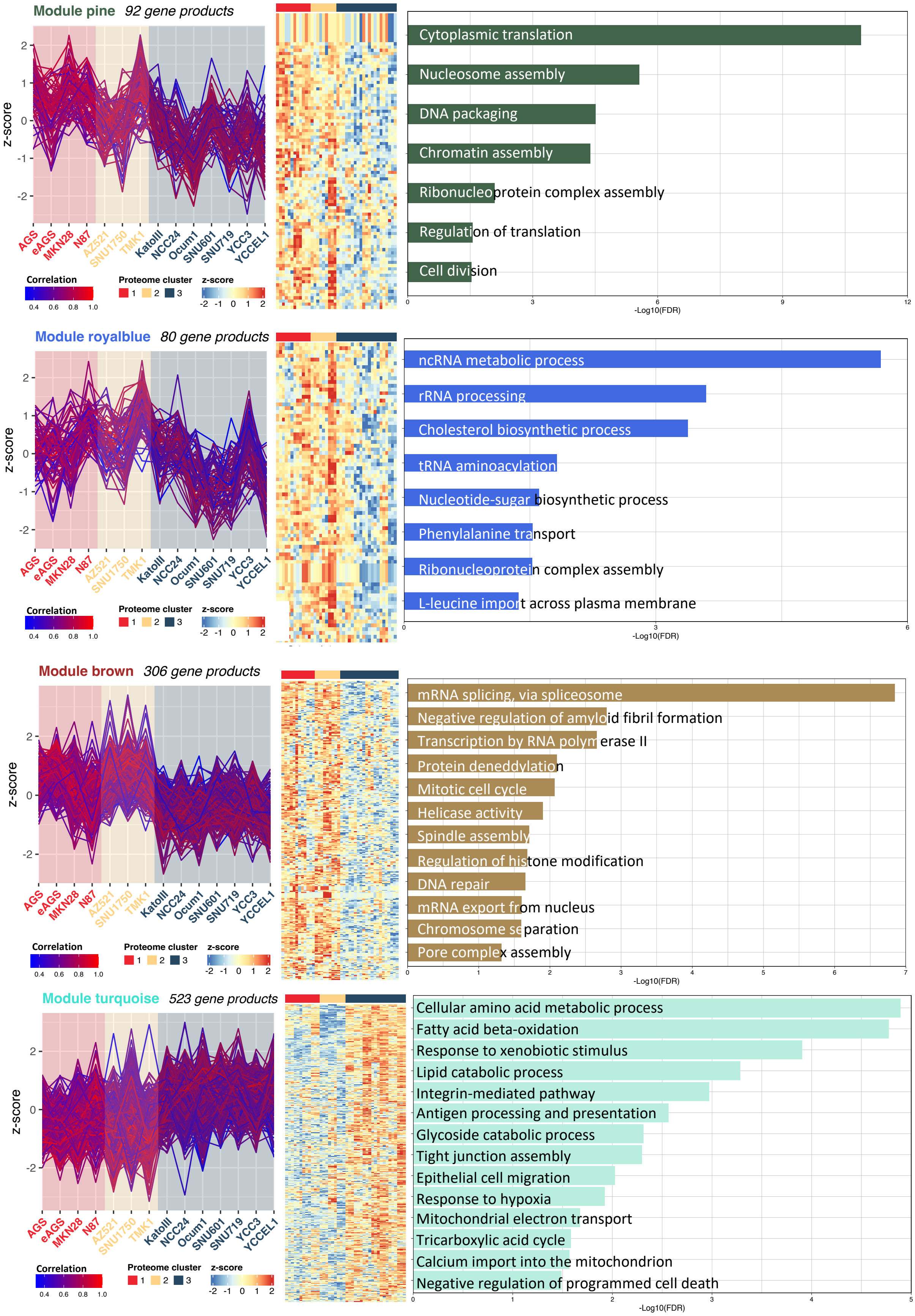

Figure S2c cont.

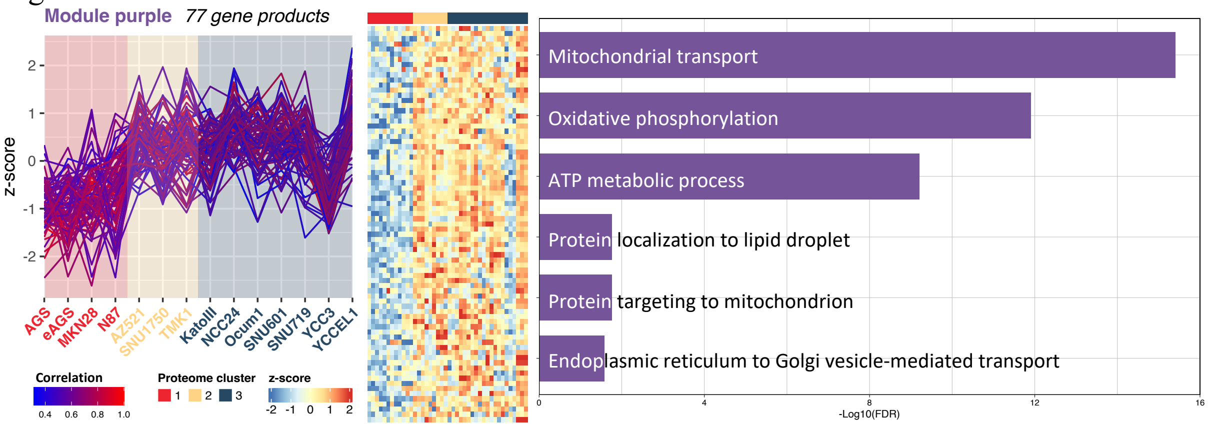

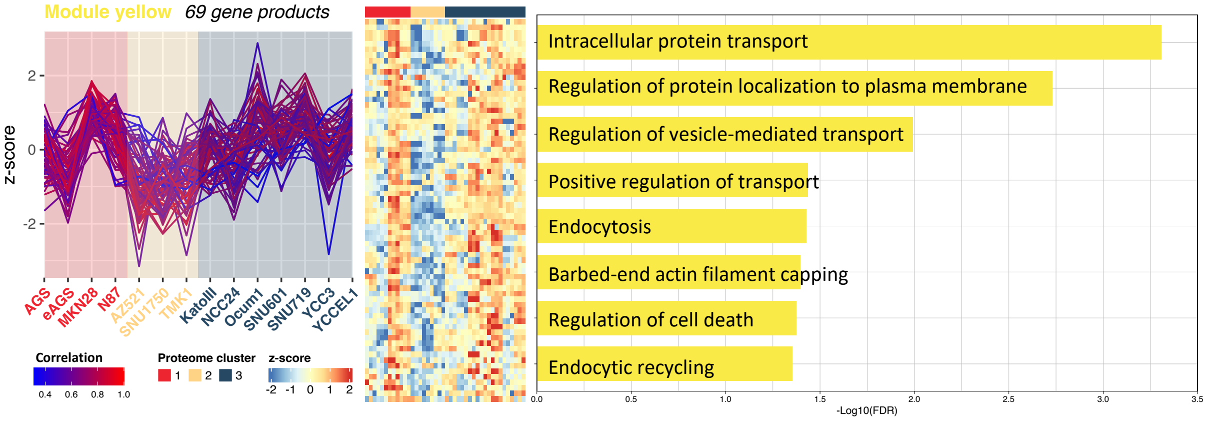

d

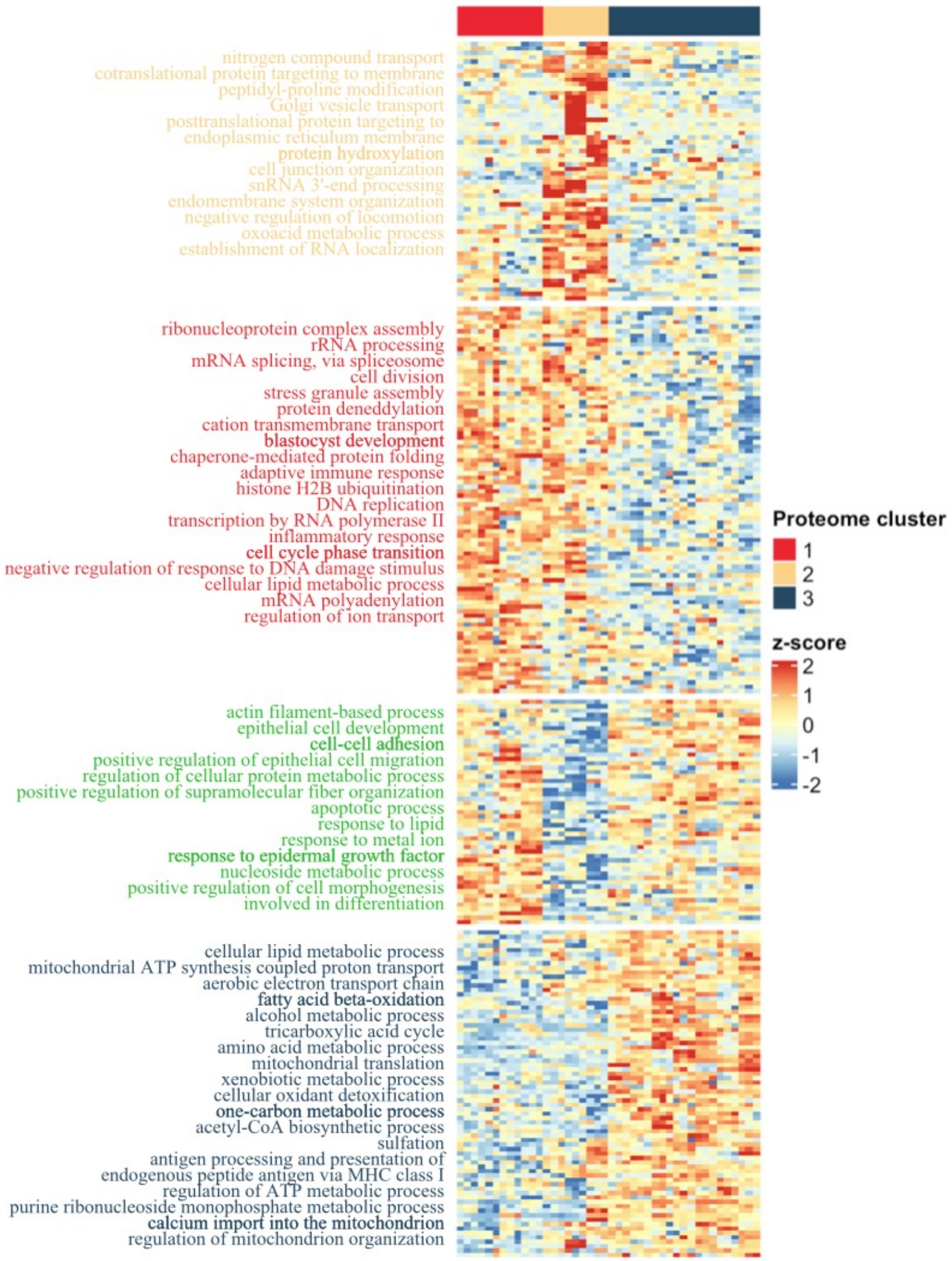

Figure S2. Subclassification based on WP and biological processes associated with each subcluster

a Cophenetic correlation coefficient plots (which measure the stability of the clusters) show how the coefficients vary with different numbers of clusters (k = 2 to 5) and different numbers of the molecules selected using percentages (10 to 100%) of median absolute deviations (MADs). The highest cophenetic coefficient was observed for a cluster number of 3 with MAD of 10%. **b** Silhouette width score plot. Silhouette scores can range from −1 to +1, where a high value indicates that the object is well matched to its own subtype and poorly matched to

neighbouring subtypes. In the best fitting of NMF results, the silhouette scores within subtypes was over 0.83 and the overall silhouette value was 0.87. **c** Functional annotation of co-expression modules. Functional annotation of nine representative colour modules are presented. Left: the co-expression patterns of the proteins in the nine modules. Middle: protein expression heatmap for the nine modules across the cell line panel. Right: representative GO terms of each module. **d** Differentially expressed proteins identified by ANOVA. The differentially expressed proteins of the three proteome clusters are presented in a heatmap (right panel) and functionally annotated by GO biological process terms on the left.

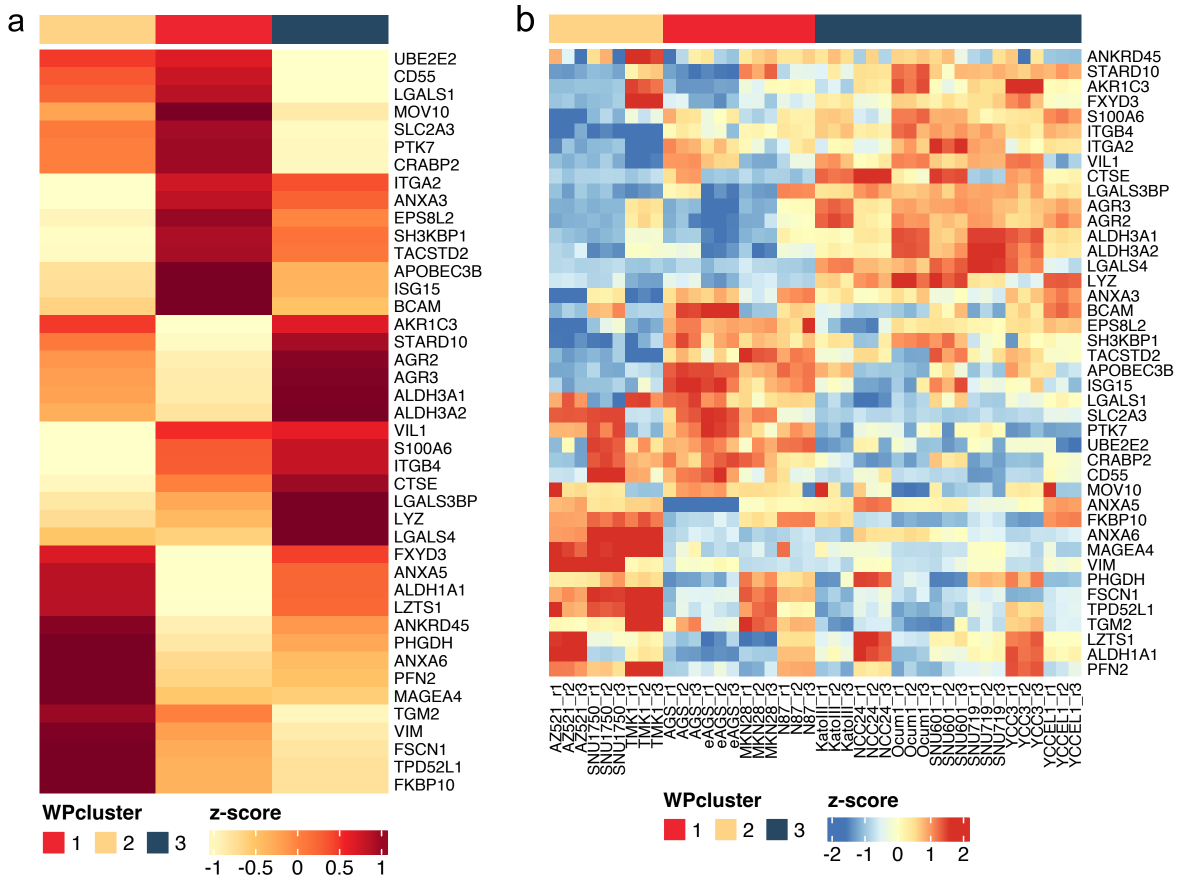

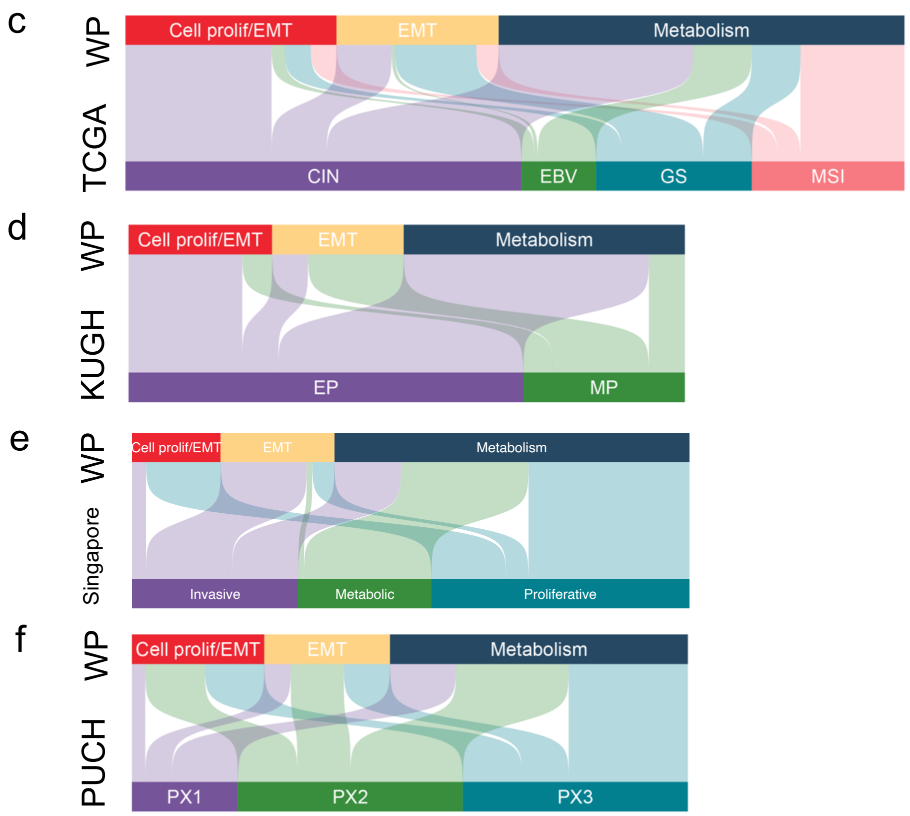

Figure S3 Metagene-specific proteins and comparison of WP subclassification with other published taxonomies

a Heatmap of the metagene matrix of WP subclassification. The most metagene-specific proteins were selected using the default method of NMF. Each column corresponds to a metagene for each WP cluster. Each row represents a protein. Rows were scaled from -1 to 1 according to the contribution of proteins to the corresponding metagene and ordered by hierarchical clustering. **b** Heatmap of the metagene composition in WP subclassification. The abundance of 42 metagene-specific proteins were scaled from -2 to 2 and plotted in the heatmap by row. GC cell line samples from three WP subgroups were plotted by column. **c-f** Comparison of WP subclassification with other GC molecular taxonomies. Comparison with TCGA subtype **(c)**, KUGH subtype **(d)**, Singapore subtype **(e)** and PUCH subtype classification **(f)**.

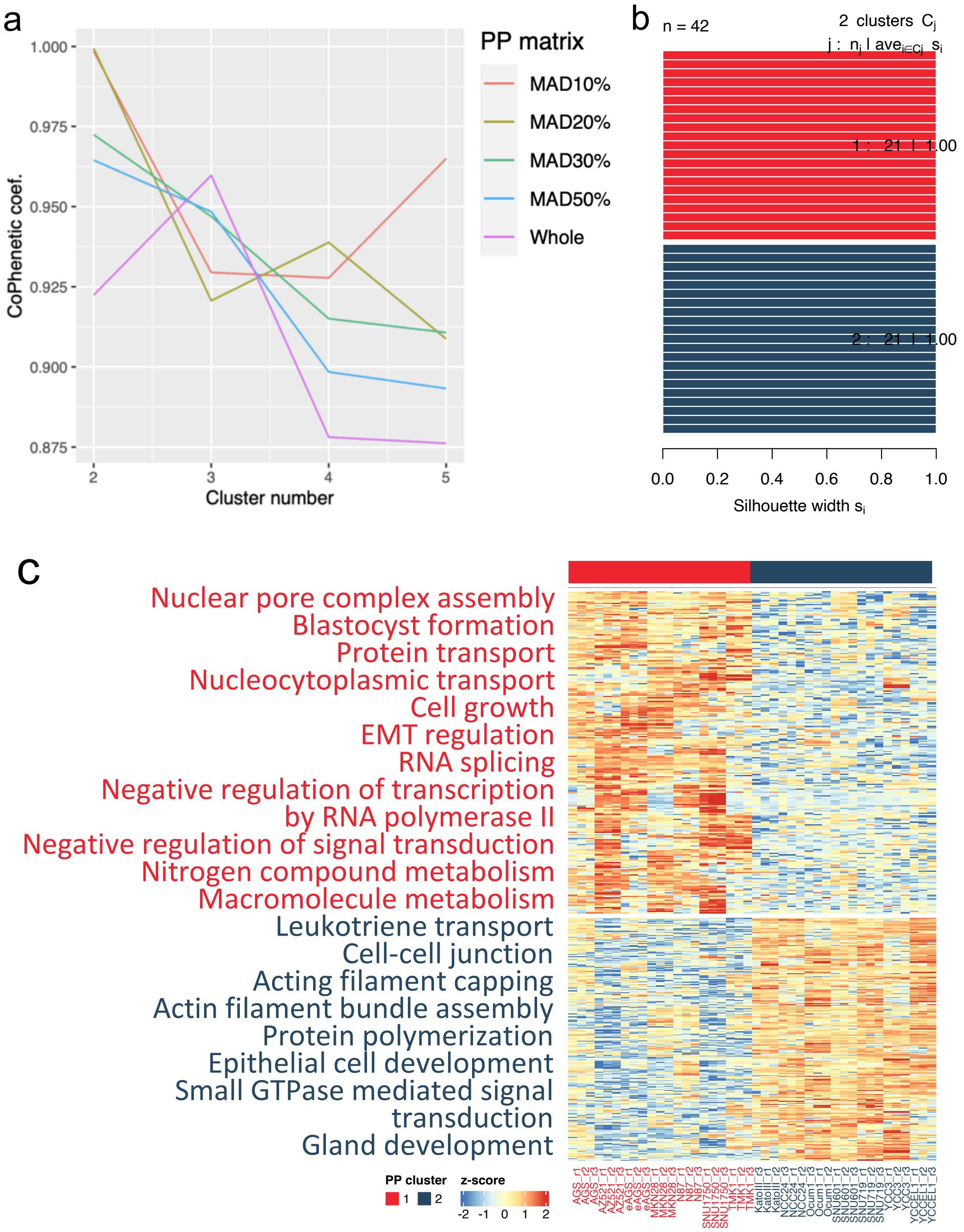
a
1.000
0.975
0.950
0.925
0.900
0.875
CoPhenetic coef.
2
3
4
5
Cluster number
PP matrix
MAD10%
MAD20%
MAD30%
MAD50%
Whole
b
n = 42
2 clusters C_j
j : n_j | $ave_{i \in Cj}$ s_i
1 : 21 | 1.00
2 : 21 | 1.00
0.0
0.2
0.4
0.6
0.8
1.0
Silhouette width s_i
c
Nuclear pore complex assembly
Blastocyst formation
Protein transport
Nucleocytoplasmic transport
Cell growth
EMT regulation
RNA splicing
Negative regulation of transcription by RNA polymerase II
Negative regulation of signal transduction
Nitrogen compound metabolism
Macromolecule metabolism
Leukotriene transport
Cell-cell junction
Acting filament capping
Actin filament bundle assembly
Protein polymerization
Epithelial cell development
Small GTPase mediated signal transduction
Gland development
PP cluster
1
2
z-score
-2 -1 0 1 2
AGS_r1
AGS_r2
AGS_r3
AZ521_r1
AZ521_r2
AZ521_r3
eAGS_r1
eAGS_r2
eAGS_r3
MKN28_r1
MKN28_r2
MKN28_r3
N87_r1
N87_r2
N87_r3
SNU1750_r1
SNU1750_r2
SNU1750_r3
TMK1_r1
TMK1_r2
TMK1_r3
KatoIII_r3
KatoIII_r1
KatoIII_r2
NCC24_r3
NCC24_r1
NCC24_r2
Ocum1_r3
Ocum1_r1
Ocum1_r2
SNU601_r1
SNU601_r2
SNU601_r3
SNU719_r1
SNU719_r2
SNU719_r3
YCC3_r1
YCC3_r2
YCC3_r3
YCCEL1_r1
YCCEL1_r2
YCCEL1_r3

d

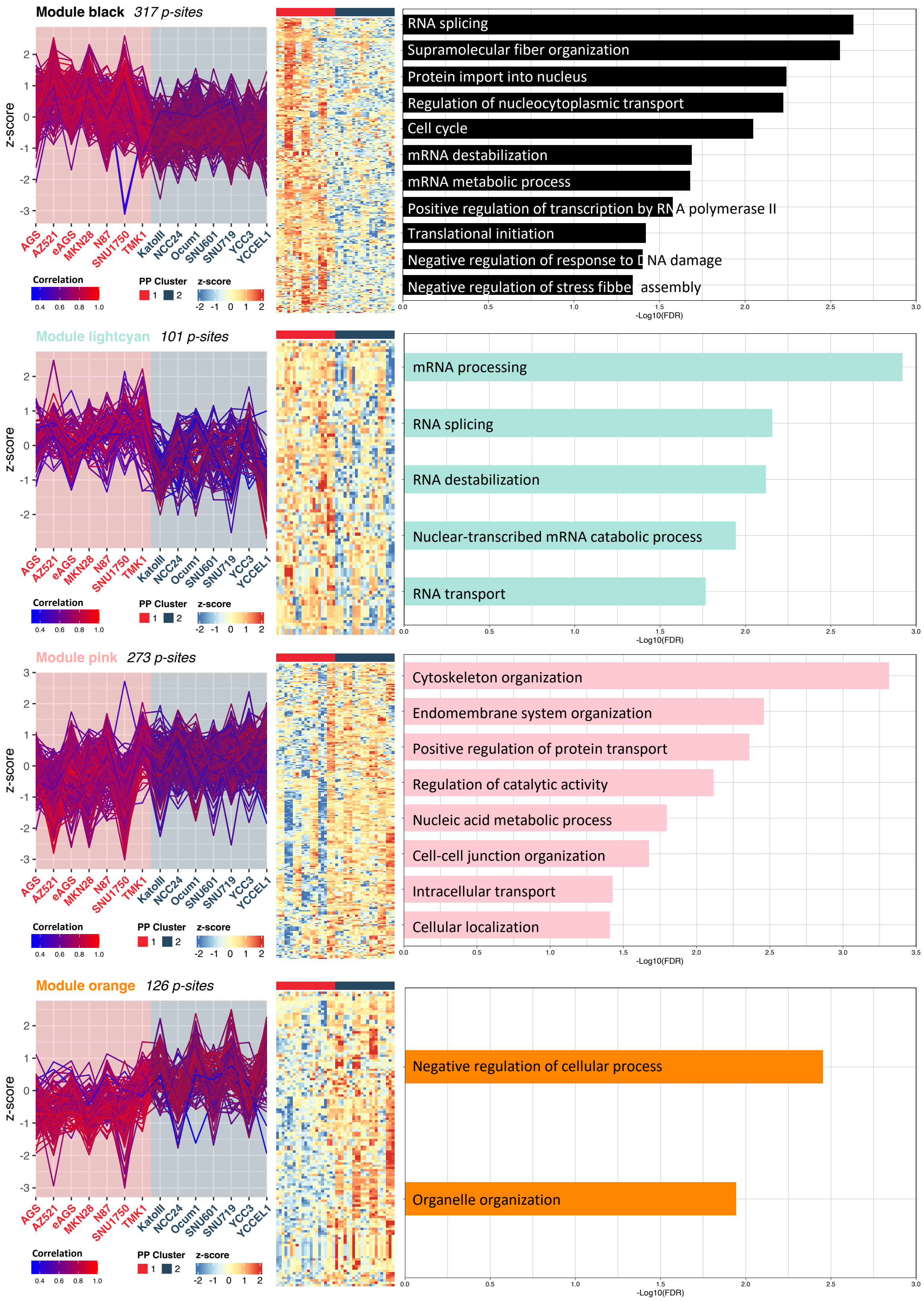

Module black 317 p-sites
z-score
AGS AZ521 eAGS MKN28 N87 SNU1750 TMK1 KatoIII NCC24 Ocum1 SNU601 SNU719 YCC3 YCCEL1
Correlation
0.4 0.6 0.8 1.0
PP Cluster 1 2
z-score -2 -1 0 1 2
RNA splicing
Supramolecular fiber organization
Protein import into nucleus
Regulation of nucleocytoplasmic transport
Cell cycle
mRNA destabilization
mRNA metabolic process
Positive regulation of transcription by RNA polymerase II
Translational initiation
Negative regulation of response to DNA damage
Negative regulation of stress fibbe assembly
-Log10(FDR)
Module lightcyan 101 p-sites
mRNA processing
RNA splicing
RNA destabilization
Nuclear-transcribed mRNA catabolic process
RNA transport
Module pink 273 p-sites
Cytoskeleton organization
Endomembrane system organization
Positive regulation of protein transport
Regulation of catalytic activity
Nucleic acid metabolic process
Cell-cell junction organization
Intracellular transport
Cellular localization
Module orange 126 p-sites
Negative regulation of cellular process
Organelle organization

Figure S4d cont.

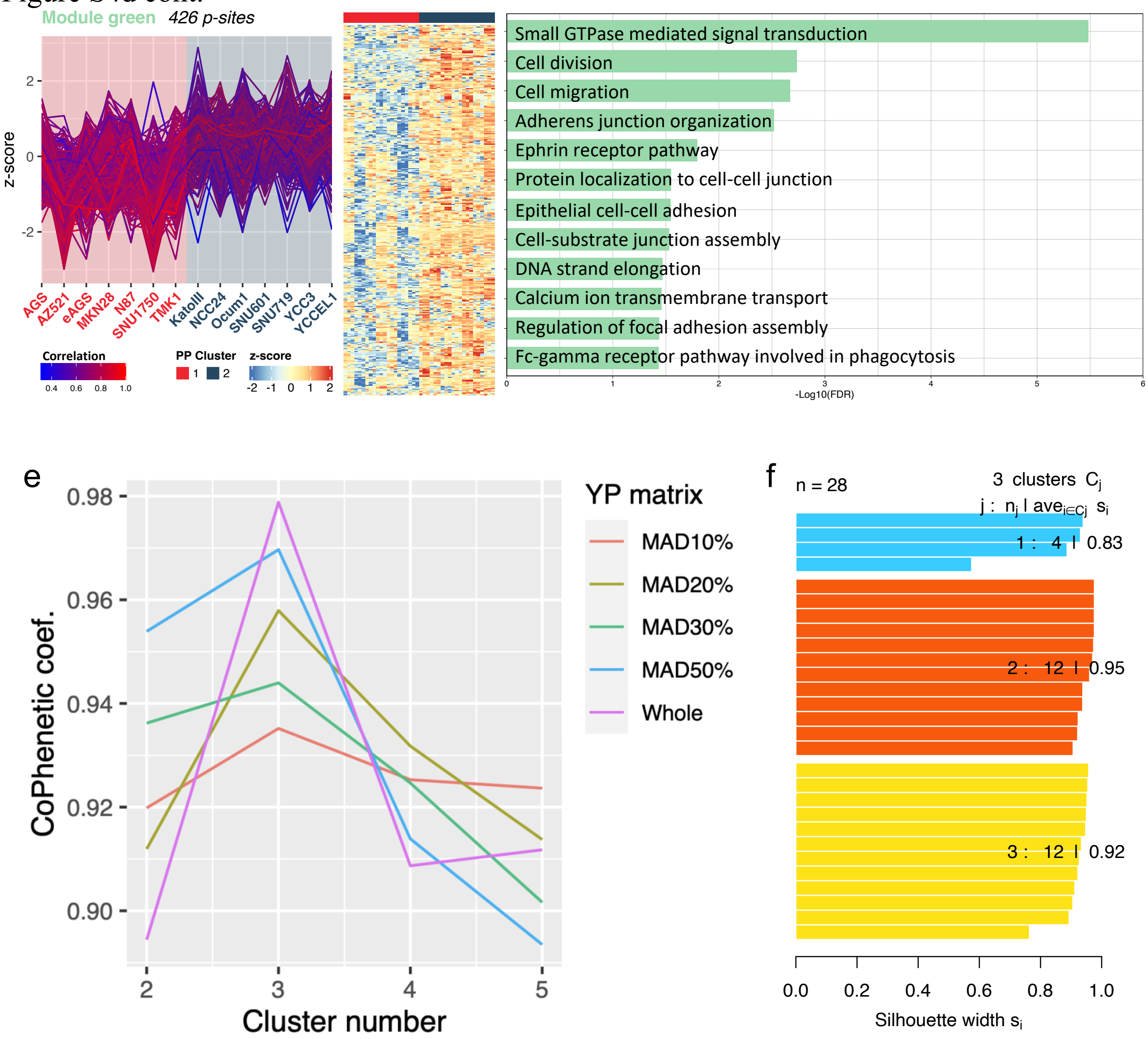

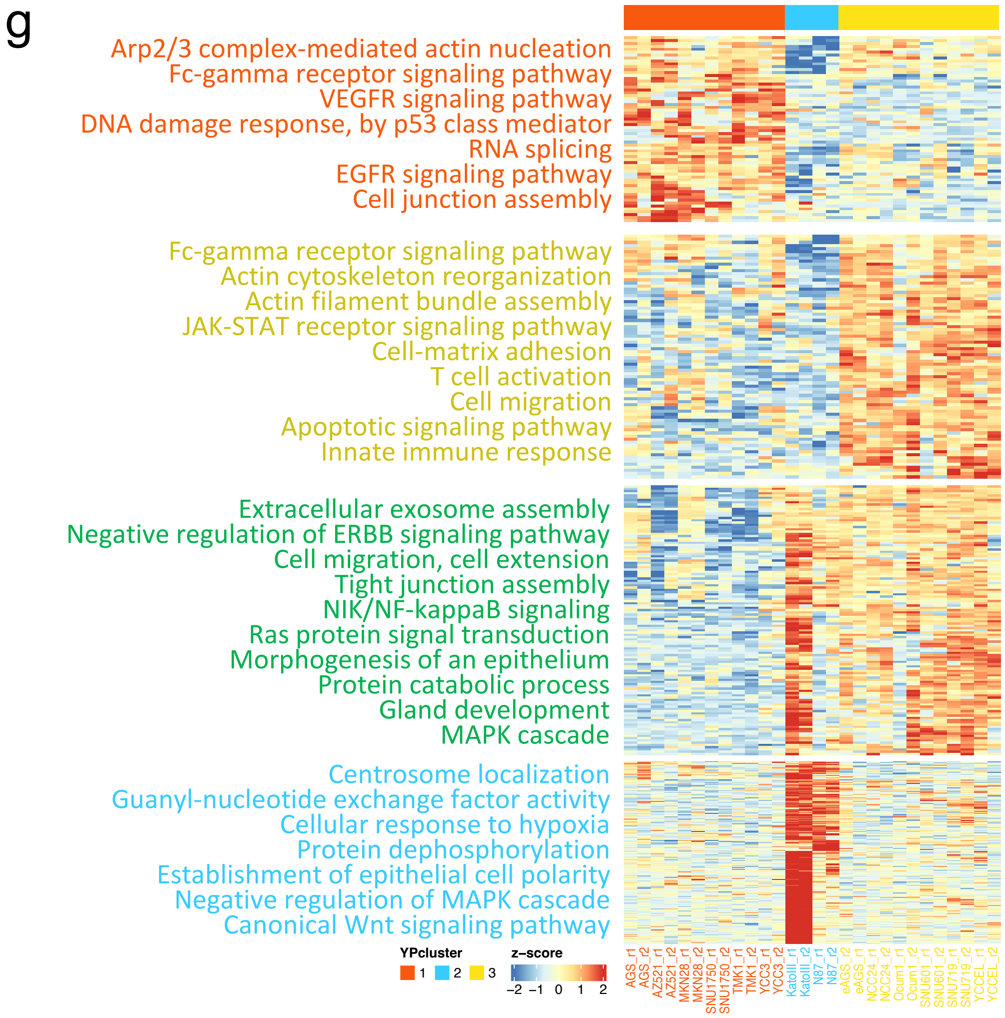

Figure S4 Subclassification based on phosphoproteomics and tyrosine phosphorylation and biological processes associated with each subcluster

a Cophenetic correlation coefficient plots for the PP dataset. **b S**ilhouette width score plot from the best fitting of NMF results. **c** Differentially expressed phosphosites and functional annotation of the PP clusters. Left panel, Representative GO terms of each subcluster; Right panel, heatmap of phosphosites in each subcluster. **d** WGCNA and gene module functional annotation in the PP clusters of GC cell lines. Functional annotation of the five colour modules in two PP clusters. Left: the co-phosphorylation patterns of the phosphosites in the five modules. Middle: phosphorylation heatmap in the five modules across the cell line panel. Right: representative GO terms for each module. **e** Cophenetic correlation coefficients for the YP dataset. **f** Silhouette width score plot from the best fitting of NMF results. **g** Differentially regulated Tyr phosphosites and functional annotation between different YP clusters. Left panel, Representative GO terms associated with each phosphosite subcluster; Right panel, Phosphorylation heatmap of phosphosites in each subcluster.

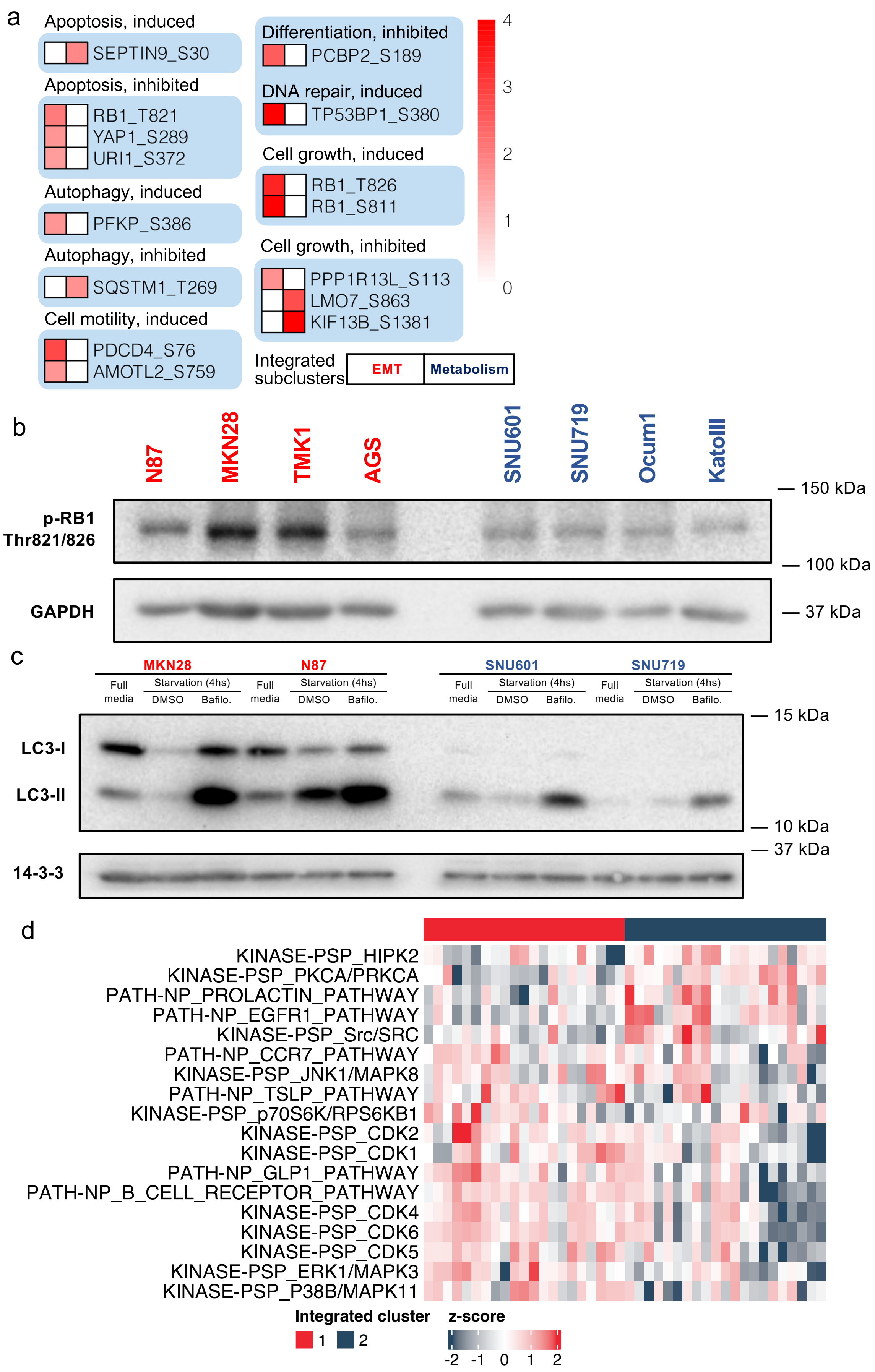
a
Apoptosis, induced
SEPTIN9_S30
Apoptosis, inhibited
RB1_T821
YAP1_S289
URI1_S372
Autophagy, induced
PFKP_S386
Autophagy, inhibited
SQSTM1_T269
Cell motility, induced
PDCD4_S76
AMOTL2_S759
Differentiation, inhibited
PCBP2_S189
DNA repair, induced
TP53BP1_S380
Cell growth, induced
RB1_T826
RB1_S811
Cell growth, inhibited
PPP1R13L_S113
LMO7_S863
KIF13B_S1381
4
3
2
1
0
Integrated subclusters
EMT
Metabolism
b
N87
MKN28
TMK1
AGS
SNU601
SNU719
Ocum1
KatoIII
150 kDa
p-RB1 Thr821/826
100 kDa
GAPDH
37 kDa
c
MKN28
N87
SNU601
SNU719
Full media
Starvation (4hs)
DMSO
Bafilo.
15 kDa
LC3-I
LC3-II
10 kDa
37 kDa
14-3-3
d
KINASE-PSP_HIPK2
KINASE-PSP_PKCA/PRKCA
PATH-NP_PROLACTIN_PATHWAY
PATH-NP_EGFR1_PATHWAY
KINASE-PSP_Src/SRC
PATH-NP_CCR7_PATHWAY
KINASE-PSP_JNK1/MAPK8
PATH-NP_TSLP_PATHWAY
KINASE-PSP_p70S6K/RPS6KB1
KINASE-PSP_CDK2
KINASE-PSP_CDK1
PATH-NP_GLP1_PATHWAY
PATH-NP_B_CELL_RECEPTOR_PATHWAY
KINASE-PSP_CDK4
KINASE-PSP_CDK6
KINASE-PSP_CDK5
KINASE-PSP_ERK1/MAPK3
KINASE-PSP_P38B/MAPK11
Integrated cluster
1
2
z-score
-2
-1
0
1
2

Figure S5 Functional annotation of phosphosites and Western blotting validation for the integrative subsets

a Differential phosphosites annotated by PhosphoSitePlus database. The red scale bar represents the ratio of phosphosite level in one versus the other integrated subset. **b** Differential expression of specific markers between the integrative subgroups. Cell lysates were harvested from eight representative cell lines, including four from the EMT subgroup (red) and four from the metabolism subgroup (dark blue). The apoptosis inhibiting phosphosite RB1_T821 exhibited increased phosphorylation in the EMT subgroup. Data are representative of n = 2 independent experiments. **c** Differential autophagy between the integrative subgroups. Cell lysates were harvested from four representative cell lines, including two from the EMT subgroup (red) and two from the metabolism subgroup (dark blue). Enhanced autophagic activity in the EMT subgroup was indicated by increased accumulation of LC3-II following 4 hours of starvation and bafilomycin A1 (Bafil.) treatment, in comparison to the metabolism subgroup. Data are representative of n = 2 independent experiments. **d** Differentially enriched signaling pathways between the integrative subgroups revealed by post-translational modifications signature enrichment analysis (PTM-SEA). Each column represents a single replicate of a particular cell line, while each row indicates the degree of enrichment for a particular pathway across all cell lines.

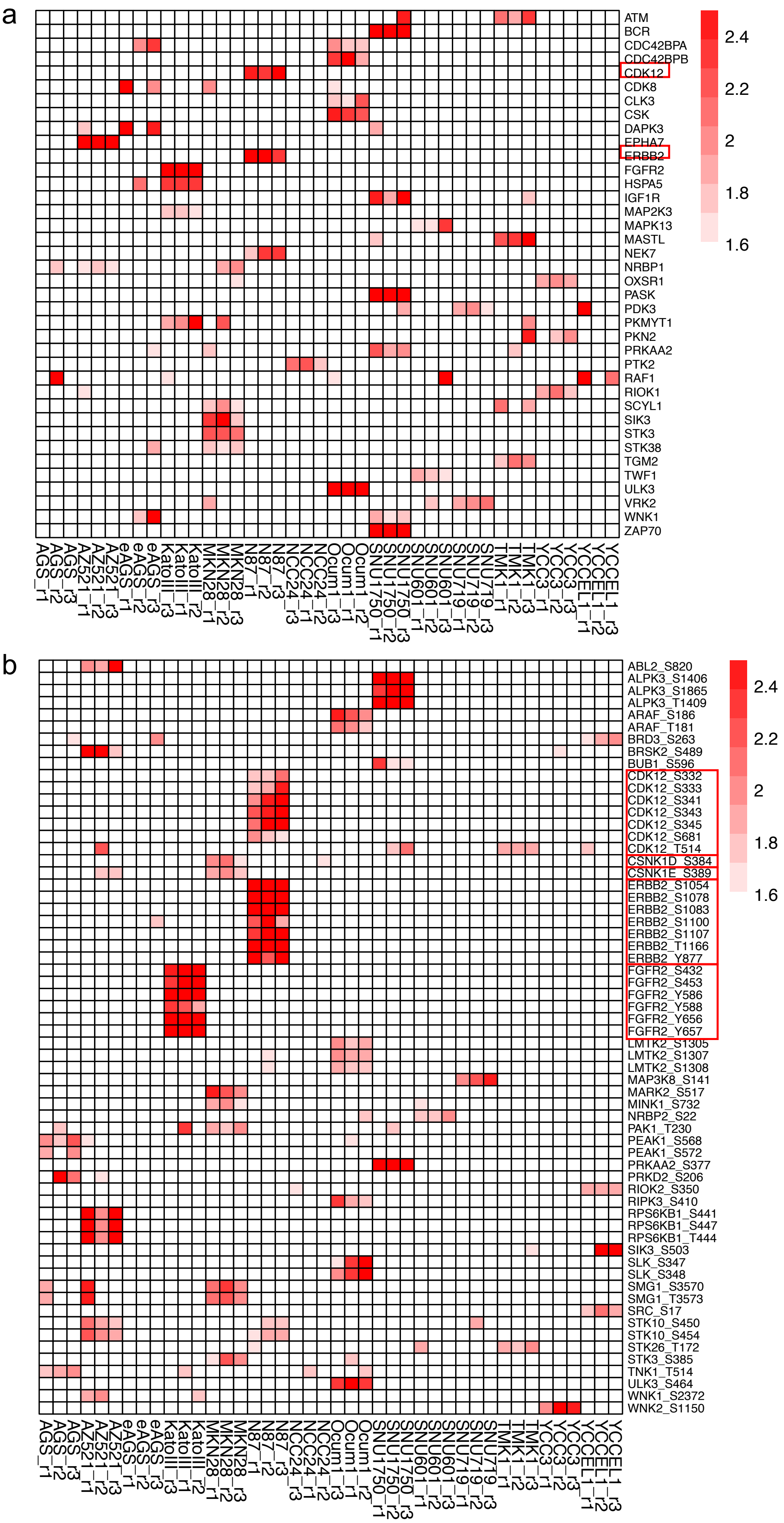
a
ATM
BCR
CDC42BPA
CDC42BPB
CDK12
CDK8
CLK3
CSK
DAPK3
EPHA7
ERBB2
FGFR2
HSPA5
IGF1R
MAP2K3
MAPK13
MASTL
NEK7
NRBP1
OXSR1
PASK
PDK3
PKMYT1
PKN2
PRKAA2
PTK2
RAF1
RIOK1
SCYL1
SIK3
STK3
STK38
TGM2
TWF1
ULK3
VRK2
WNK1
ZAP70
2.4
2.2
2
1.8
1.6
AGS_r1
AGS_r2
AGS_r3
AZ521_r1
AZ521_r2
AZ521_r3
eAGS_r1
eAGS_r2
eAGS_r3
KatoIII_r1
KatoIII_r2
KatoIII_r3
MKN28_r1
MKN28_r2
MKN28_r3
N87_r1
N87_r2
N87_r3
NCC24_r1
NCC24_r2
NCC24_r3
Ocum1_r1
Ocum1_r2
Ocum1_r3
SNU1750_r1
SNU1750_r2
SNU1750_r3
SNU601_r1
SNU601_r2
SNU601_r3
SNU719_r1
SNU719_r2
SNU719_r3
TMK1_r1
TMK1_r2
TMK1_r3
YCC3_r1
YCC3_r2
YCC3_r3
YCCEL1_r1
YCCEL1_r2
YCCEL1_r3
b
ABL2_S820
ALPK3_S1406
ALPK3_S1865
ALPK3_T1409
ARAF_S186
ARAF_T181
BRD3_S263
BRSK2_S489
BUB1_S596
CDK12_S332
CDK12_S333
CDK12_S341
CDK12_S343
CDK12_S345
CDK12_S681
CDK12_T514
CSNK1D_S384
CSNK1E_S389
ERBB2_S1054
ERBB2_S1078
ERBB2_S1083
ERBB2_S1100
ERBB2_S1107
ERBB2_T1166
ERBB2_Y877
FGFR2_S432
FGFR2_S453
FGFR2_Y586
FGFR2_Y588
FGFR2_Y656
FGFR2_Y657
LMTK2_S1305
LMTK2_S1307
LMTK2_S1308
MAP3K8_S141
MARK2_S517
MINK1_S732
NRBP2_S22
PAK1_T230
PEAK1_S568
PEAK1_S572
PRKAA2_S377
PRKD2_S206
RIOK2_S350
RIPK3_S410
RPS6KB1_S441
RPS6KB1_S447
RPS6KB1_T444
SIK3_S503
SLK_S347
SLK_S348
SMG1_S3570
SMG1_T3573
SRC_S17
STK10_S450
STK10_S454
STK26_T172
STK3_S385
TNK1_T514
ULK3_S464
WNK1_S2372
WNK2_S1150
2.4
2.2
2
1.8
1.6
AGS_r1
AGS_r2
AGS_r3
AZ521_r1
AZ521_r2
AZ521_r3
eAGS_r1
eAGS_r2
eAGS_r3
KatoIII_r1
KatoIII_r2
KatoIII_r3
MKN28_r1
MKN28_r2
MKN28_r3
N87_r1
N87_r2
N87_r3
NCC24_r1
NCC24_r2
NCC24_r3
Ocum1_r1
Ocum1_r2
Ocum1_r3
SNU1750_r1
SNU1750_r2
SNU1750_r3
SNU601_r1
SNU601_r2
SNU601_r3
SNU719_r1
SNU719_r2
SNU719_r3
TMK1_r1
TMK1_r2
TMK1_r3
YCC3_r1
YCC3_r2
YCC3_r3
YCCEL1_r1
YCCEL1_r2
YCCEL1_r3

c

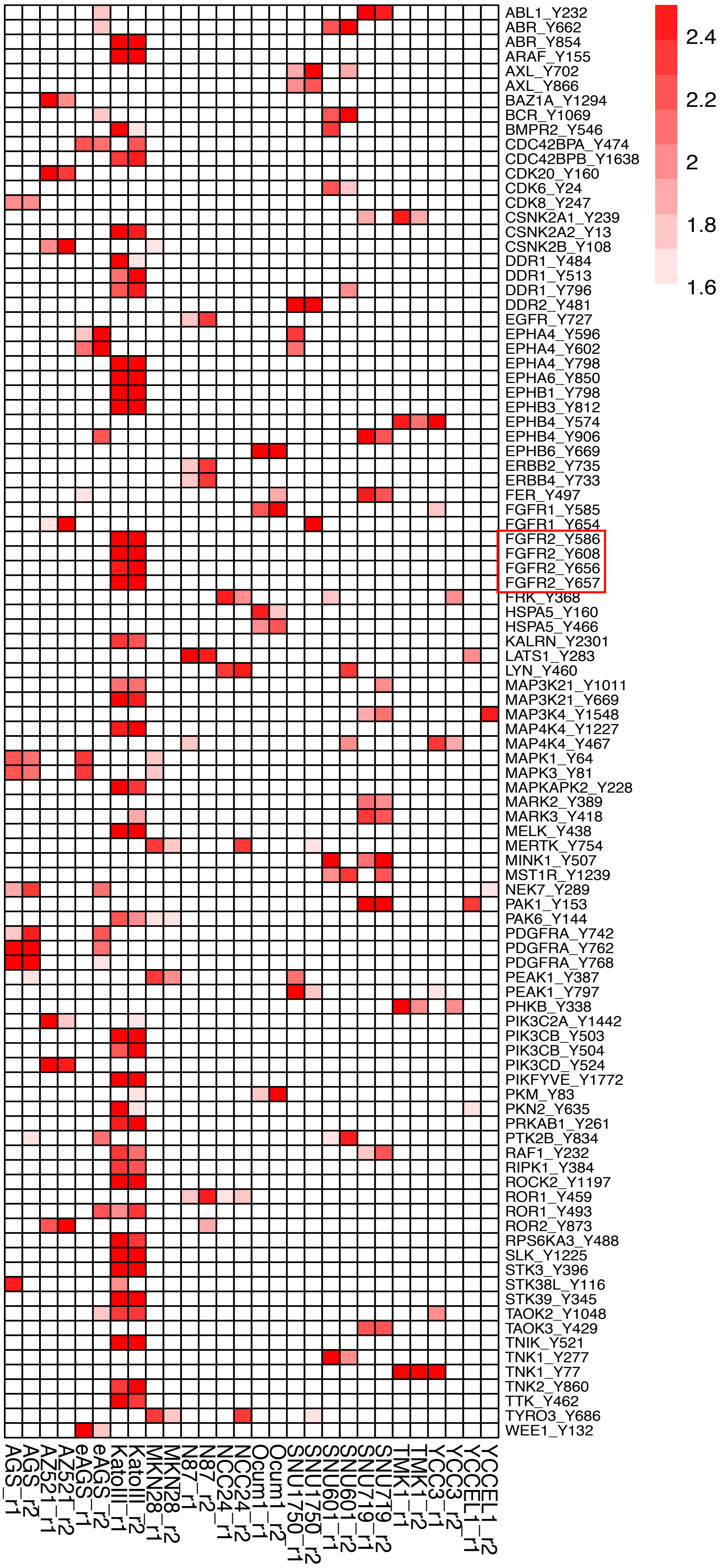
ABL1_Y232
ABR_Y662
ABR_Y854
ARAF_Y155
AXL_Y702
AXL_Y866
BAZ1A_Y1294
BCR_Y1069
BMPR2_Y546
CDC42BPA_Y474
CDC42BPB_Y1638
CDK20_Y160
CDK6_Y24
CDK8_Y247
CSNK2A1_Y239
CSNK2A2_Y13
CSNK2B_Y108
DDR1_Y484
DDR1_Y513
DDR1_Y796
DDR2_Y481
EGFR_Y727
EPHA4_Y596
EPHA4_Y602
EPHA4_Y798
EPHA6_Y850
EPHB1_Y798
EPHB3_Y812
EPHB4_Y574
EPHB4_Y906
EPHB6_Y669
ERBB2_Y735
ERBB4_Y733
FER_Y497
FGFR1_Y585
FGFR1_Y654
FGFR2_Y586
FGFR2_Y608
FGFR2_Y656
FGFR2_Y657
FRK_Y368
HSPA5_Y160
HSPA5_Y466
KALRN_Y2301
LATS1_Y283
LYN_Y460
MAP3K21_Y1011
MAP3K21_Y669
MAP3K4_Y1548
MAP4K4_Y1227
MAP4K4_Y467
MAPK1_Y64
MAPK3_Y81
MAPKAPK2_Y228
MARK2_Y389
MARK3_Y418
MELK_Y438
MERTK_Y754
MINK1_Y507
MST1R_Y1239
NEK7_Y289
PAK1_Y153
PAK6_Y144
PDGFRA_Y742
PDGFRA_Y762
PDGFRA_Y768
PEAK1_Y387
PEAK1_Y797
PHKB_Y338
PIK3C2A_Y1442
PIK3CB_Y503
PIK3CB_Y504
PIK3CD_Y524
PIKFYVE_Y1772
PKM_Y83
PKN2_Y635
PRKAB1_Y261
PTK2B_Y834
RAF1_Y232
RIPK1_Y384
ROCK2_Y1197
ROR1_Y459
ROR1_Y493
ROR2_Y873
RPS6KA3_Y488
SLK_Y1225
STK3_Y396
STK38L_Y116
STK39_Y345
TAOK2_Y1048
TAOK3_Y429
TNIK_Y521
TNK1_Y277
TNK1_Y77
TNK2_Y860
TTK_Y462
TYRO3_Y686
WEE1_Y132
AGS_r1
AGS_r2
AZ521_r1
AZ521_r2
eAGS_r1
eAGS_r2
KatoIII_r1
KatoIII_r2
MKN28_r1
MKN28_r2
N87_r1
N87_r2
NCC24_r1
NCC24_r2
Ocum1_r1
Ocum1_r2
SNU1750_r1
SNU1750_r2
SNU601_r1
SNU601_r2
SNU719_r1
SNU719_r2
TMK1_r1
TMK1_r2
YCC3_r1
YCC3_r2
YCCEL1_r1
YCCEL1_r2
2.4
2.2
2
1.8
1.6

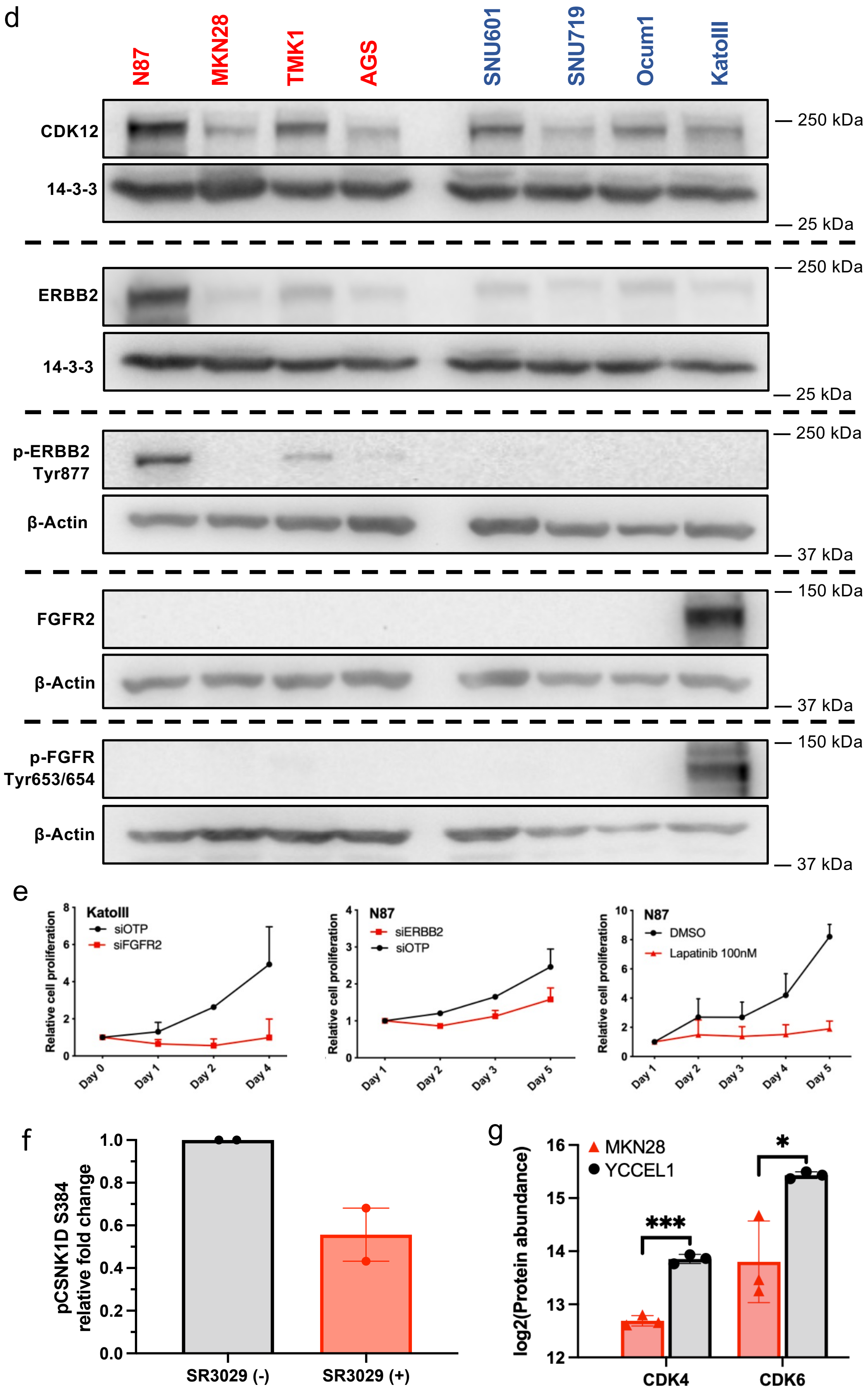

d
N87
MKN28
TMK1
AGS
SNU601
SNU719
Ocum1
KatoIII
CDK12
14-3-3
ERBB2
p-ERBB2
Tyr877
β-Actin
FGFR2
p-FGFR
Tyr653/654
— 250 kDa
— 25 kDa
— 37 kDa
— 150 kDa
e
KatoIII
siOTP
siFGFR2
N87
siERBB2
DMSO
Lapatinib 100nM
Relative cell proliferation
Day 0
Day 1
Day 2
Day 3
Day 4
Day 5
f
pCSNK1D S384
relative fold change
SR3029 (-)
SR3029 (+)
g
MKN28
YCCEL1
log2(Protein abundance)
CDK4
CDK6
***
*

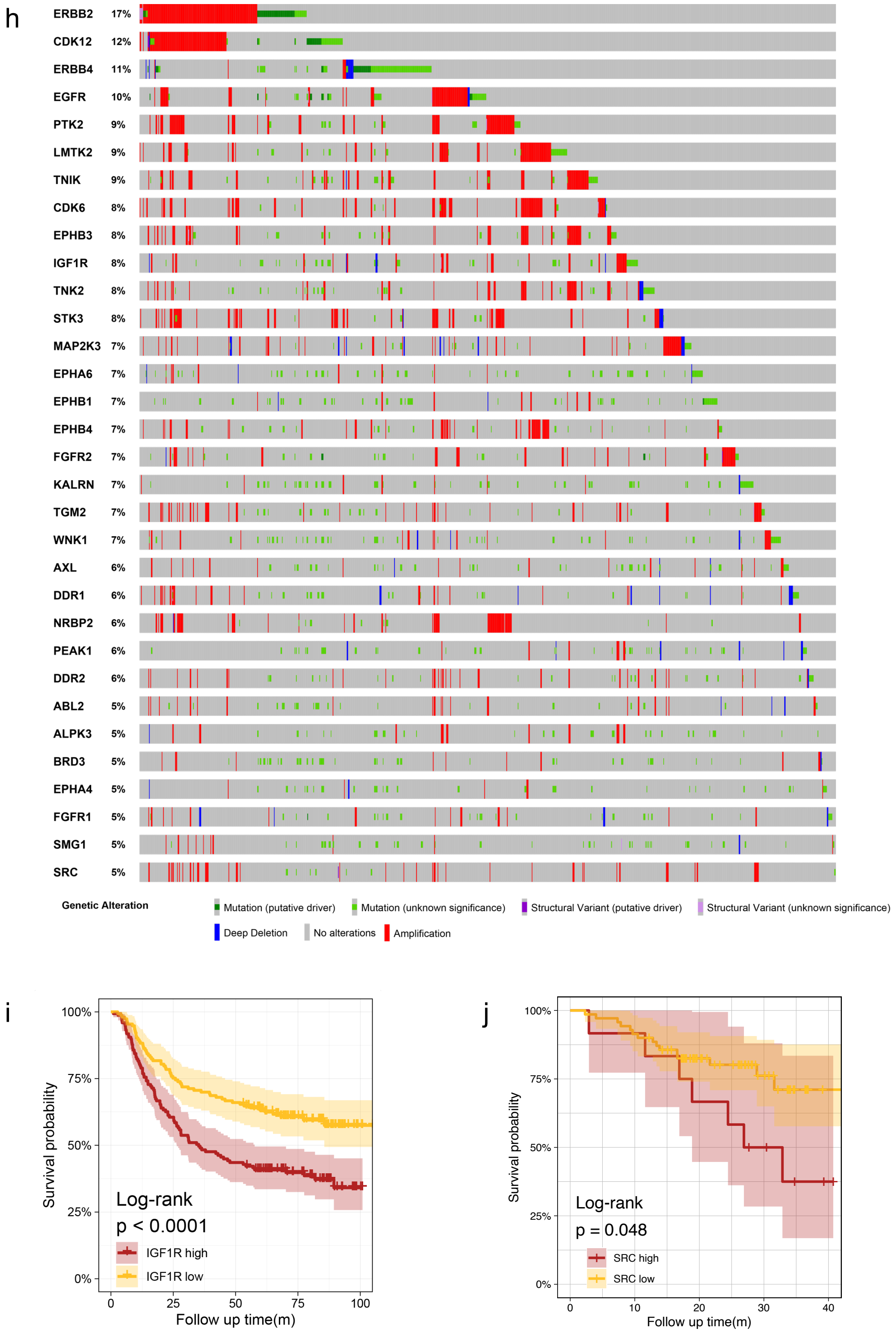

Figure S6 Outlier kinases in the GC cell line panel and GC patient cohort

a-c Outlier kinases of the WP (A), PP (B) and YP (C) datasets. Kinases with outlying high intensities are highlighted with the red shading. **d** Validation of expression of specific outliers across the cell line panel. Cell lysates were harvested from specific GC cell lines. Phosphorylated and/or total forms of CDK12, FGFR2 and ERBB2 were significantly elevated in specific GC cell lines as predicted by the MS analysis. Data are representative of n = 2 independent experiments. **e** MTS assay following selective kinase knockdown or inhibitor

treatment in the corresponding GC cell lines. Data are expressed relative to day 1, which was arbitrarily set at 1. Error bars represent the range of the mean from n=2 independent experiments. si: small interfering RNA; siOTP: ON-TARGETplus^{TM} small interfering RNA. **f** Decreased phosphorylation at CSNK1D Ser384 in MKN28 cells after SR3029 treatment. Data represent the mean and variation of two independent MS-based phosphoproteomics experiments using DDA and DIA modes respectively. **g** CDK4/6 expression in the MKN28 and YCCEL1 cell lines. Protein expression data of CDK4/6 were extracted from WP data and compared between MKN28 and YCCEL1 cell lines. *, P<0.05; ***, P<0.001. **h** Genomic alterations in outlier kinases across 1512 GC patients. Outlier kinases with mutation or amplification in at least 5% of GC patients are depicted using Oncoprint by cBioPortal. Patients without mutation/amplification of these 32 outlier kinases were removed. Percentages on the left of the graph show the percentage of total DNA alterations in each outlier kinase across the GC cohort. **i** Kaplan-Meier survival analysis based on expression of the outlier kinase IGF1R at the mRNA level. **j** Kaplan-Meier survival analysis based on expression of the outlier kinase SRC at the protein level.

a

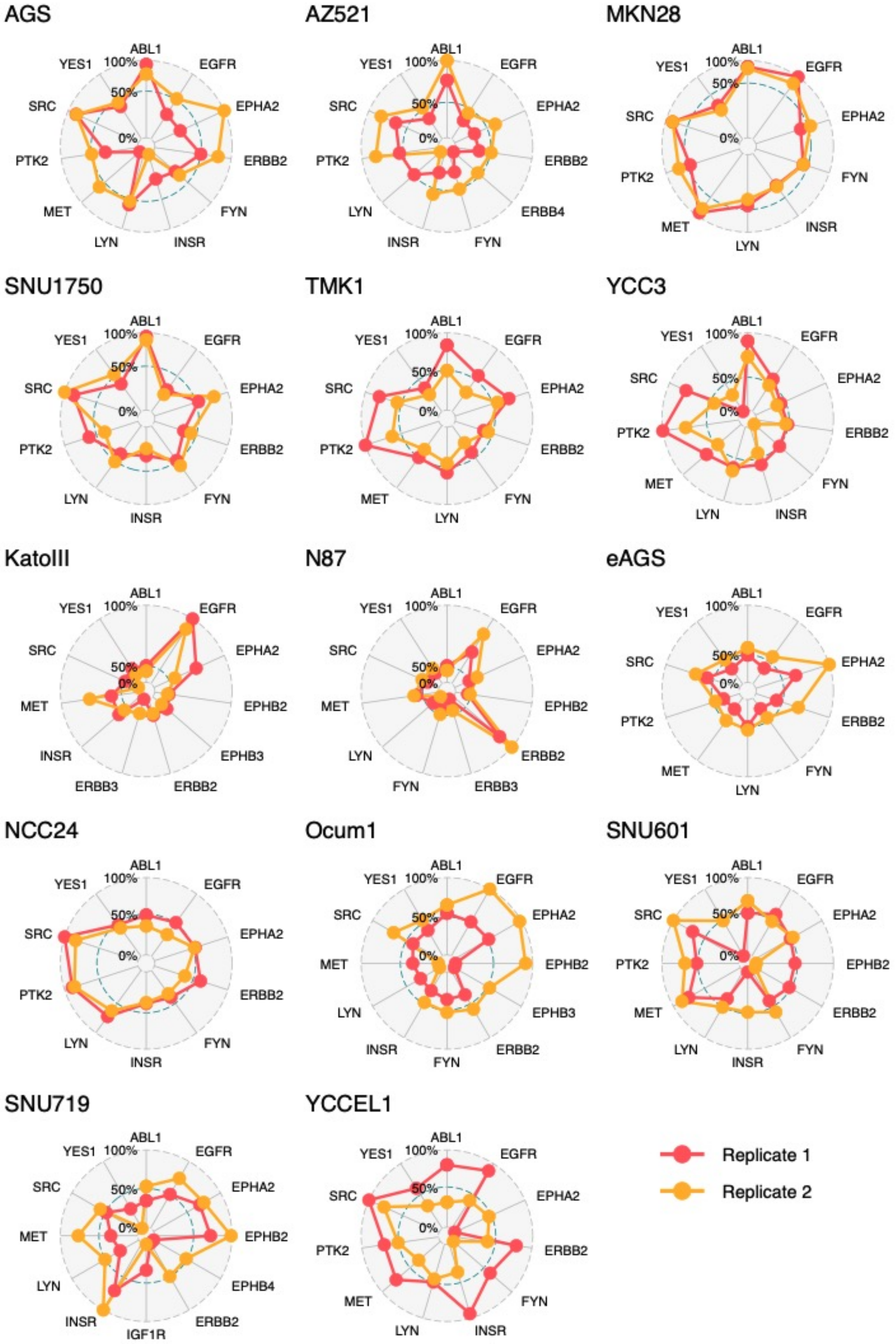
AGS
ABL1
EGFR
EPHA2
ERBB2
FYN
INSR
LYN
MET
PTK2
SRC
YES1
100%
50%
0%
AZ521
ABL1
EGFR
EPHA2
ERBB2
ERBB4
FYN
INSR
LYN
PTK2
SRC
YES1
MKN28
ABL1
EGFR
EPHA2
FYN
INSR
LYN
MET
PTK2
SRC
YES1
SNU1750
ABL1
EGFR
EPHA2
ERBB2
FYN
INSR
LYN
PTK2
SRC
YES1
TMK1
ABL1
EGFR
EPHA2
ERBB2
FYN
LYN
MET
PTK2
SRC
YES1
YCC3
ABL1
EGFR
EPHA2
ERBB2
FYN
INSR
LYN
MET
PTK2
SRC
YES1
KatoIII
ABL1
EGFR
EPHA2
EPHB2
EPHB3
ERBB2
ERBB3
INSR
MET
SRC
YES1
N87
ABL1
EGFR
EPHA2
EPHB2
ERBB2
ERBB3
FYN
LYN
MET
SRC
YES1
eAGS
ABL1
EGFR
EPHA2
ERBB2
FYN
LYN
MET
PTK2
SRC
YES1
NCC24
ABL1
EGFR
EPHA2
ERBB2
FYN
INSR
LYN
PTK2
SRC
YES1
Ocum1
ABL1
EGFR
EPHA2
EPHB2
EPHB3
ERBB2
FYN
INSR
LYN
MET
SRC
YES1
SNU601
ABL1
EGFR
EPHA2
EPHB2
ERBB2
FYN
INSR
LYN
MET
PTK2
SRC
YES1
SNU719
ABL1
EGFR
EPHA2
EPHB2
EPHB4
ERBB2
IGF1R
INSR
LYN
MET
SRC
YES1
YCCEL1
ABL1
EGFR
EPHA2
ERBB2
FYN
INSR
LYN
MET
PTK2
SRC
YES1
Replicate 1
Replicate 2

b

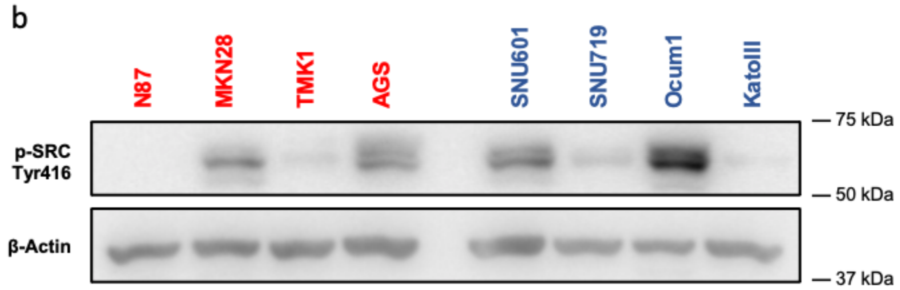

Figure S7. Tyrosine kinase activity in GC cell lines

a Each axis of the radar plot represents one of the top activated tyrosine kinases in a specific cell line inferred from INKA scoring. The further towards the edge of the spoke a point reaches, the higher the kinase activity. **b** The phosphorylation of SRC Tyr416 was interrogated by Western blotting across GC cell lines. Data are representative of n = 2 independent experiments. The same gel was used for blotting FGFR2 (Figure S6d) as well as p-SRC, hence they share the same loading control.

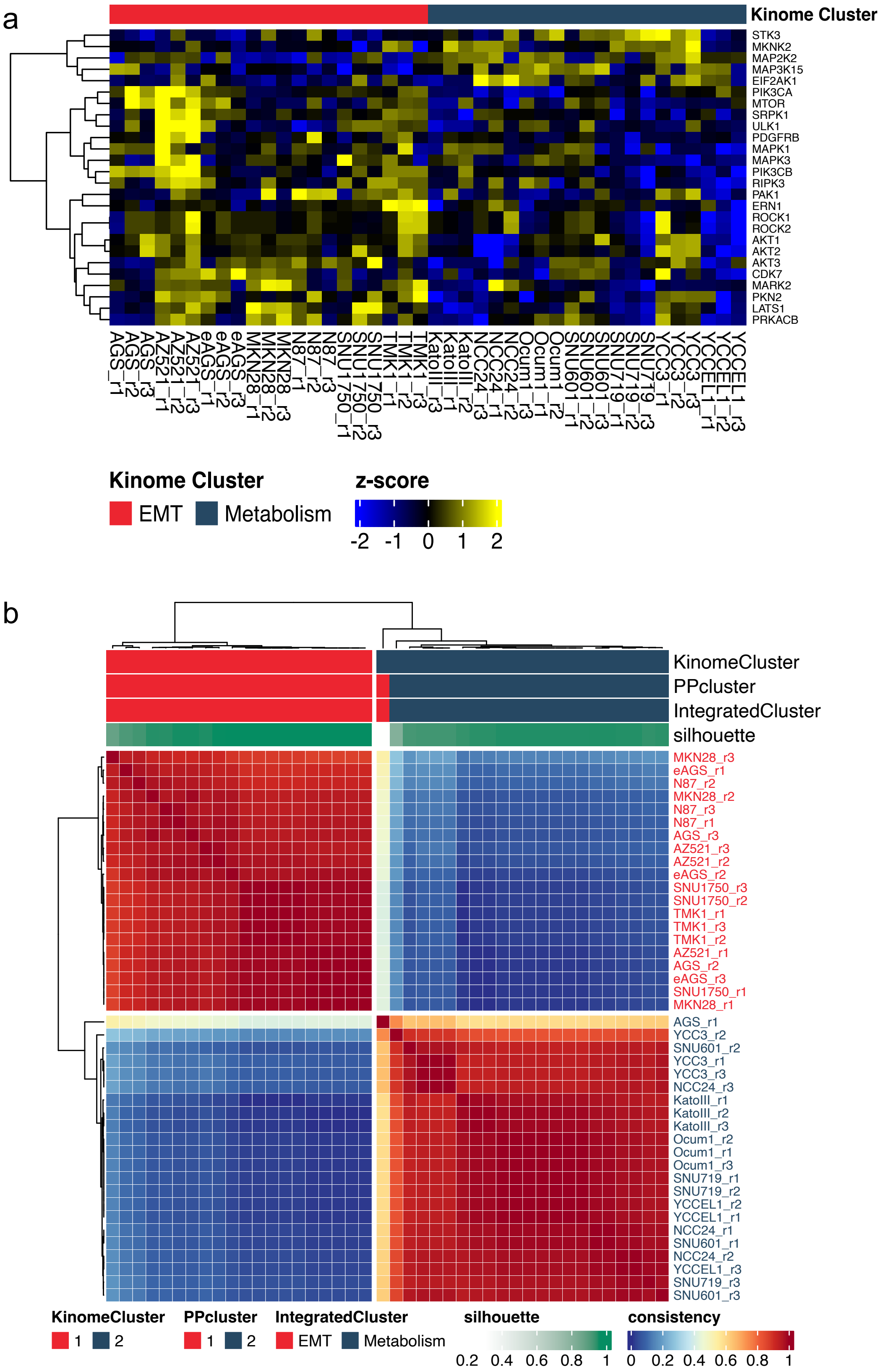

a
Kinome Cluster
STK3
MKNK2
MAP2K2
MAP3K15
EIF2AK1
PIK3CA
MTOR
SRPK1
ULK1
PDGFRB
MAPK1
MAPK3
PIK3CB
RIPK3
PAK1
ERN1
ROCK1
ROCK2
AKT1
AKT2
AKT3
CDK7
MARK2
PKN2
LATS1
PRKACB
AGS_r1
AGS_r2
AGS_r3
AZ521_r1
AZ521_r2
AZ521_r3
eAGS_r1
eAGS_r2
eAGS_r3
MKN28_r1
MKN28_r2
MKN28_r3
N87_r1
N87_r2
N87_r3
SNU1750_r1
SNU1750_r2
SNU1750_r3
TMK1_r1
TMK1_r2
TMK1_r3
KatoIII_r1
KatoIII_r2
KatoIII_r3
NCC24_r1
NCC24_r2
NCC24_r3
Ocum1_r1
Ocum1_r2
Ocum1_r3
SNU601_r1
SNU601_r2
SNU601_r3
SNU719_r1
SNU719_r2
SNU719_r3
YCC3_r1
YCC3_r2
YCC3_r3
YCCEL1_r1
YCCEL1_r2
YCCEL1_r3
Kinome Cluster
EMT
Metabolism
z-score
-2 -1 0 1 2
b
KinomeCluster
PPcluster
IntegratedCluster
silhouette
MKN28_r3
eAGS_r1
N87_r2
MKN28_r2
N87_r3
N87_r1
AGS_r3
AZ521_r3
AZ521_r2
eAGS_r2
SNU1750_r3
SNU1750_r2
TMK1_r1
TMK1_r3
TMK1_r2
AZ521_r1
AGS_r2
eAGS_r3
SNU1750_r1
MKN28_r1
AGS_r1
YCC3_r2
SNU601_r2
YCC3_r1
YCC3_r3
NCC24_r3
KatoIII_r1
KatoIII_r2
KatoIII_r3
Ocum1_r2
Ocum1_r1
Ocum1_r3
SNU719_r1
SNU719_r2
YCCEL1_r2
YCCEL1_r1
NCC24_r1
SNU601_r1
NCC24_r2
YCCEL1_r3
SNU719_r3
SNU601_r3
KinomeCluster
1 2
PPcluster
1 2
IntegratedCluster
EMT Metabolism
silhouette
0.2 0.4 0.6 0.8 1
consistency
0 0.2 0.4 0.6 0.8 1

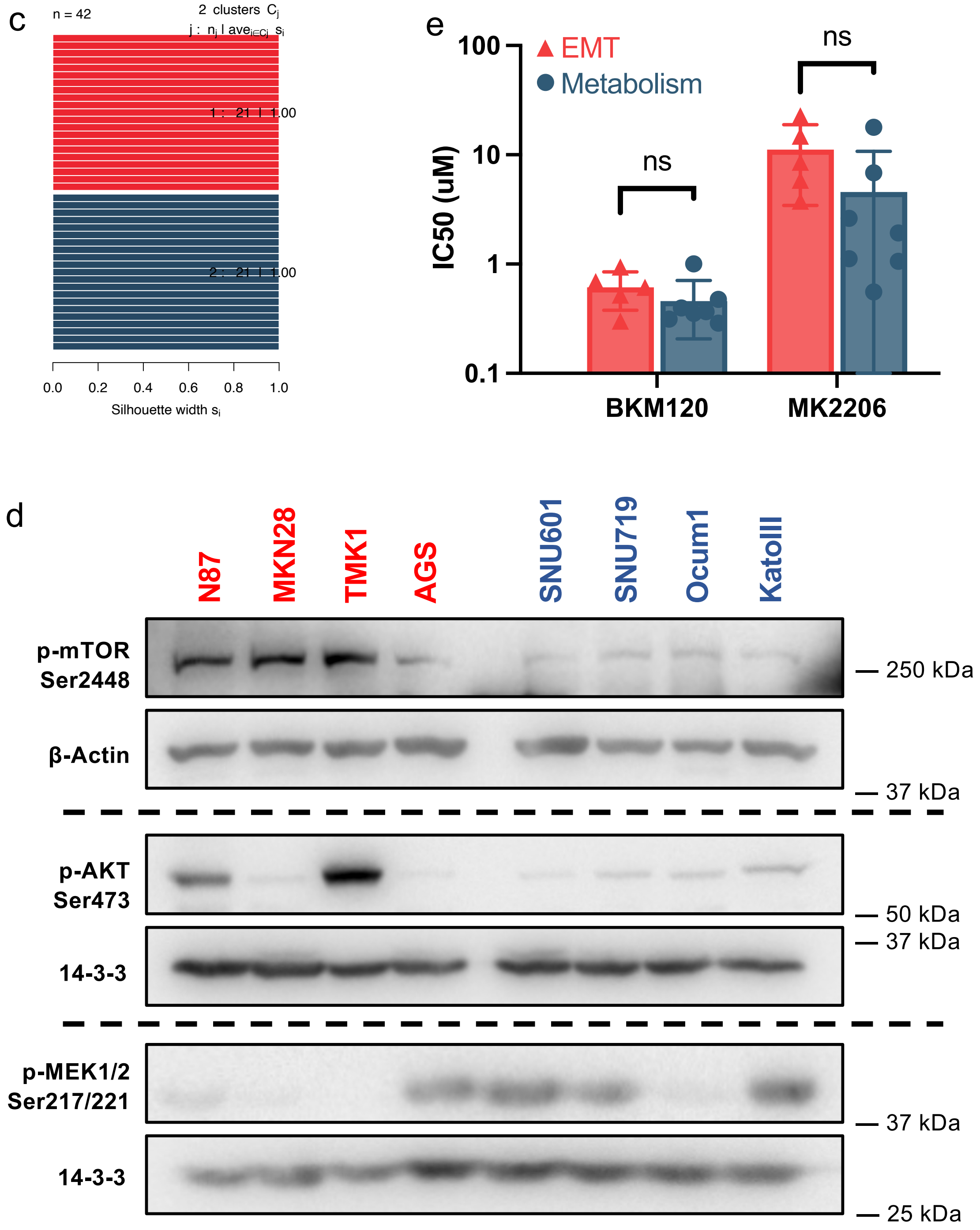

Figure S8 Kinomic subclassification and group specific vulnerabilities of GC cell line panel

a Predicted differentially-activated kinases between the EMT and metabolism subgroups. The heatmap represents output obtained using the KSEA algorithm using normalized PP. Six activity matrices were used in total (see Methods). Heatmaps show kinases (rows) arranged by hierarchical clustering and kinase activity level for each kinase. Yellow represents high kinase activity whereas navy blue represents low activation level. Subclassification of the cell lines is shown at the top. **b** NMF classification using the WP kinome in GC cell lines. The heatmap presents the sample consensus obtained from NMF clustering, with the blue-to-red gradient denoting the consistency of the clustering results. The silhouette score of each sample after 100 times of NMF clustering is indicated with a green-white gradient. **c**. Silhouette width score plot

from the best fitting of NMF results. Both similarity within subtypes and the overall silhouette value reached 1.0. **d** Differential phosphorylation of mTOR_S2448, AKT_S473 and MAP2K1/2_S217/221 between the EMT and metabolism subgroups. Cell lysates were harvested from representative cell lines of the EMT subgroup (red) and metabolism subgroup (dark blue) and subjected to Western blotting as indicated. Data are representative of n = 2 independent experiments. The same gels were used for blotting ERBB2 (Figure S6d) and pAkt (Figure S8d), and FGFR2 (Figure S6d), p-SRC (Figure S7b) and p-mTOR (Figure S8d), hence they share the same loading controls. **e** IC50 values of BKM120 and MK2206 for inhibition of GC cell lines in the EMT and metabolism subgroups. The IC50 value was derived from dose response analysis using a MTS assay.
